# Generating Long-Lived Triplet Excited States in Narrow Bandgap Conjugated Polymers

**DOI:** 10.1021/jacs.2c12008

**Published:** 2023-02-03

**Authors:** Jose M. Marin-Beloqui, Daniel G. Congrave, Daniel T. W. Toolan, Stephanie Montanaro, Junjun Guo, Iain A. Wright, Tracey M. Clarke, Hugo Bronstein, Stoichko D. Dimitrov

**Affiliations:** † Department of Chemistry, University College London, London WC1H 0AJ, U.K.; ‡ Department of Physical-Chemistry, University of Málaga, Campus de Teatinos, Málaga, 29071 Málaga, Spain; § Department of Chemistry, University of Cambridge, Cambridge CB2 1EW, U.K.; ∥ Department of Chemistry, Dainton Building, The University of Sheffield, Brook Hill, Sheffield S3 7HF, U.K.; ⊥ Department of Chemistry, Loughborough University, Loughborough LE11 3TU, U.K.; # School of Chemistry, University of Edinburgh, Edinburgh EH9 3FJ, U.K.; ∇ Department of Chemistry, Queen Mary University of London, London E1 4NS, U.K.

## Abstract

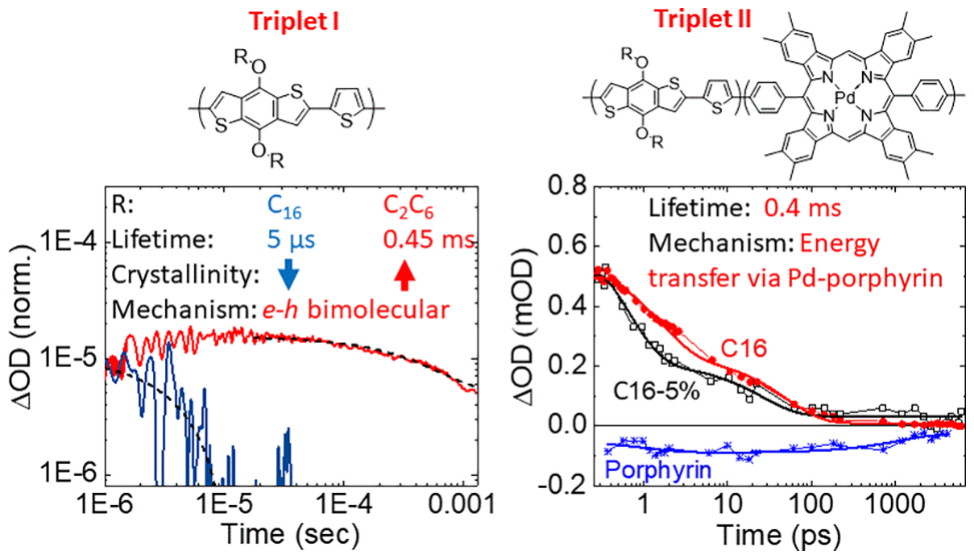

Narrow bandgap conjugated polymers are a heavily studied class of organic semiconductors, but their excited states usually have a very short lifetime, limiting their scope for applications. One approach to overcome the short lifetime is to populate long-lived triplet states for which relaxation to the ground state is forbidden. However, the triplet lifetime of narrow bandgap polymer films is typically limited to a few microseconds. Here, we investigated the effect of film morphology on triplet dynamics in red-emitting conjugated polymers based on the classic benzodithiophene monomer unit with the solubilizing alkyl side chains C_16_ and C_2_C_6_ and then used Pd porphyrin sensitization as a further strategy to change the triplet dynamics. Using transient absorption spectroscopy, we demonstrated a 0.45 ms triplet lifetime for the more crystalline nonsensitized polymer C_2_C_6_, 2–3 orders of magnitude longer than typically reported, while the amorphous C_16_ had only a 5 μs lifetime. The increase is partly due to delaying bimolecular electron–hole recombination in the more crystalline C_2_C_6,_ where a higher energy barrier for charge recombination is expected. A triplet lifetime of 0.4 ms was also achieved by covalently incorporating 5% of Pd porphyrin into the C_16_ polymer, which introduced extra energy transfer steps between the polymer and porphyrin that delayed triplet dynamics and increased the polymer triplet yield by 7.9 times. This work demonstrates two synthetic approaches to generate the longest-lived triplet excited states in narrow bandgap conjugated polymers, which is of necessity in a wide range of fields that range from organic electronics to sensors and bioapplications.

## Introduction

A great effort has been dedicated to exploiting narrow bandgap conjugated polymers, which can absorb light across the visible spectrum and into the near-infrared, to develop them for photovoltaic, photodetector, and sensing applications.^1^ In them, the excited-state lifetime (τ) is a fundamental figure of merit. Large excited-state lifetimes correlate to a reduction in nonradiative decay^2,3^ (the single most important efficiency loss pathway in organic photovoltaic (OPV) and photodetectors) and an increase of the exciton diffusion length toward more reproducible optoelectronic device architectures. However, in the solid state, most narrow bandgap conjugated polymers have singlet excited states with very short lifetimes of tens to hundreds of picoseconds, e.g., ca. 30 ps for the record OPV polymer PM6.^4^ Short lifetimes are often connected to intermolecular interactions inducing fast nonradiative relaxation pathways to the ground state,^5^ but are also related more fundamentally to the energy gap law.^6,7^ One rational way to increase the excited-state lifetime of narrow bandgap polymers is to populate lower-energy triplet (T) excited states rather than relying on the singlet (S) states formed upon photoexcitation. In contrast to singlets, triplets are excited states with a net spin multiplicity of one. Hence, T*_n_* → S_0_ conversion is formally spin-forbidden, fundamentally increasing the lifetime of triplet excited states compared to singlets, e.g., the triplet lifetime of the polymer PTB7 is 0.8 μs, while the singlet lifetime is only 93 ± 48 ps.^7,8^

Triplets are usually formed via intersystem crossing from the photoexcited singlet. Strong spin–orbit coupling (SOC) and favorable S–T energy alignment promotes higher triplet yields, but it can also lead to shorter triplet lifetime by enabling T*_n_* → S_0_ conversion. It is also possible to triplet-sensitize polymers using metal–porphyrin complexes, which can have 100% intersystem crossing yield, thereby elongating the polymer excited-state lifetime significantly due to the introduction of intermediate energy transfer steps between the polymer and metal–porphyrin excited states.^9−11^ There are other possible mechanisms for triplet generation. In organic heterojunctions, triplets can be formed via bimolecular or monomolecular recombination of photogenerated electrons and holes on the nanosecond to microsecond timescales.^12−14^ Bimolecular electron–hole recombination follows spin statistics and creates 3/4 triplets from all recombination events. Similarly, in molecular crystals, long-lived triplets have been generated at donor–acceptor interfaces utilizing charge-transfer states, which has allowed room-temperature phosphorescence to be achieved.^15^

Here, we synthesized two red-emitting conjugated polymers, implementing benzodithiophene (BDT) and thiophene (T) units, which are ubiquitous in state-of-the-art OPV,^16^ photocatalytic,^17^ and photodetector^18^ materials (e.g., PM6, PTB7, and D18).^19−21^ Two different solubilizing alkyl chains, branched (C_2_C_6_) and linear (C_16_), were attached to the BDT-T core to cause changes in thin-film crystallinity. Transient absorption (TA) spectroscopy on the picosecond to millisecond timescales was used to assess the impact of film order on the triplet generation dynamics. The results identify that long-lived triplets are formed in both polymer films, but the more crystalline C_2_C_6_ polymer has a much longer triplet lifetime of 0.45 ms compared to the 5 μs of the more amorphous C_16_ polymer. Their triplet formation times also differ: from tens of microseconds for C_2_C_6_ to sub-microsecond for C_16_, which is caused by slower rates of bimolecular recombination in the more crystalline C_2_C_6_ polymer. This difference is likely due to a larger energy barrier for charge recombination due to a greater proportion of crystalline regions in the C_2_C_6_ film.^22,23^ As a second step in this work, we sensitized the C_16_ polymers covalently with Pd porphyrin to increase the triplet yield and number of relaxation steps in the excited-state dynamics. The result was a larger triplet yield and lifetime increase to 0.4 ms, which is again extremely long for such conjugated polymers in comparison with others found in the literature.^8,24,25^

## Experimental Section

The synthetic details, thin-film fabrication, and chemical analyses of the materials are provided in the Supporting Information (SI). Microsecond TAS was measured using a 6 ns, 10 Hz Nd:YAG laser (Spectra-Physics, INDI-40-10) as the excitation source. The excitation wavelength was generated with a versaScan L-532 OPO. The excitation density was changed from 1 to 400 μJ/cm^2^ with neutral density filters and measured with an ES111C photometer (Thorlabs). A quartz tungsten halogen lamp (IL1, Bentham) was used as a probe light. TA signals were acquired with Si and InGaAs photodiodes coupled to a preamplifier and an electronic filter (Costronic Electronics) connected to an oscilloscope (Tektronix DPO4034B) and PC. Probe wavelengths were selected with a monochromator (Cornerstone 130, Oriel Instruments) before the detector. During measurements, samples were kept under a controlled atmosphere with a sealed cuvette to either nitrogen or oxygen flow.

Picosecond to nanosecond transient absorption spectroscopy was carried out using Solstice regenerative amplifier (Newport corporation) with 800 nm pump pulses and operating at 1 kHz. The output beam is split into two parts to seed a TOPAS-NIRUVIS instrument generating the pump pulses and a transient absorption spectrometer equipped with visible and near-infrared detection (Helios, Ultrafast systems). All experiments were carried out with thin films kept under constant nitrogen flow. No material degradation was observed during experiments. Data were analyzed using Python, Excel, Surface Xplorer, and Origin. Global analysis was carried out with the free-source Optimus software.^26^

## Results and Discussion

**Figure 1:**
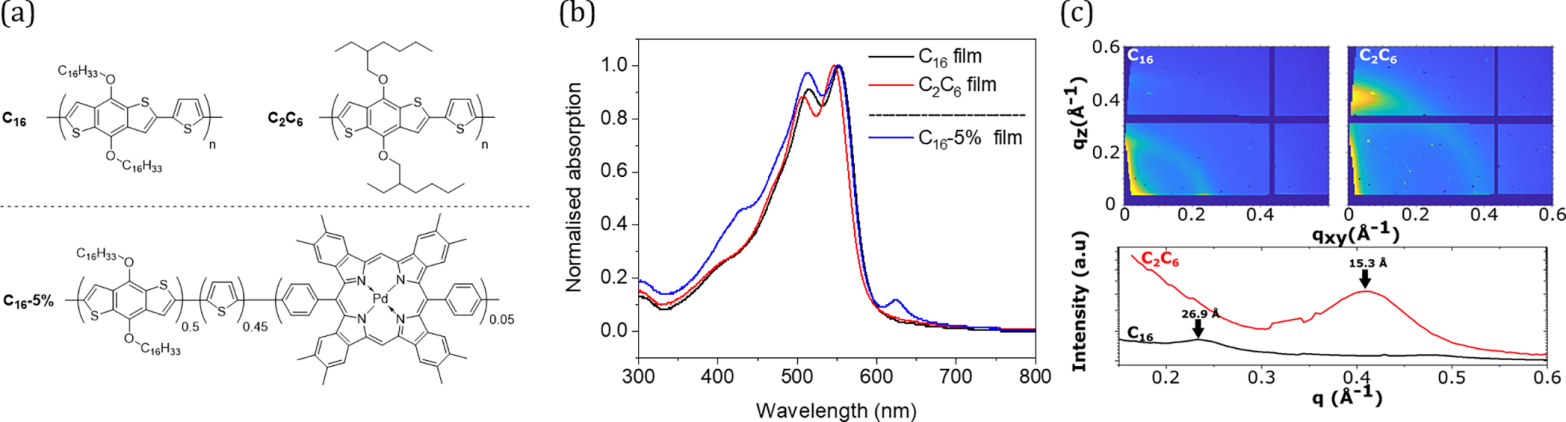
(a) Chemical structures of the investigated polymers. (b) Thin-film absorption spectra. (c) Thin-film X-ray diffraction data.

In this study, the popular building block BDT is coupled to thiophene to obtain the red-emitting conjugated polymer, PBDT-T.^27^ The polymers studied here are hereafter referred to by their solubilizing chains: linear hexadecyl (C_16_) and branched 2-ethyl(hexyl) (C_2_C_6_), which were varied to access films with differing morphologies. The structures of the studied materials are shown in Figure 1a. As the generation of long-lived triplet states is the main target in this study, a C_16_ polymer with an extended Pd porphyrin incorporated into the conjugated backbone at 5 mol % loading (C_16_-5%) was also synthesized to investigate the effect of the metal porphyrin’s ultrafast and efficient intersystem crossing.^11^ From the onset of the UV–vis spectrum, the S_1_ energy is estimated to be 2.05 and 2.0 eV for C_2_C_6_ and C_16_ (C_16_-5%), respectively.^28^ X-ray diffraction data reveal significant differences between the pristine polymers of C_16_ and C_2_C_6_ (Figure 1c). Stronger and sharper diffraction peaks are observed for C_2_C_6_, clearly indicating a more ordered film structure compared to C_16_. The materials show 100 lamellae features at 15 and 27 Å for the C_2_C_6_ and C_16_ films, respectively. For the C_2_C_6_, this is entirely consistent with no interdigitation of the branched alkyl side chains. For the linear C_16_, the feature size is 27 Å, while we would expect a single linear C_16_ to be ∼18 Å, so the linear alkyl chains are either interdigitated or are not fully extended from the thiophene core. The second explanation seems more plausible as if the C_16_ were interdigitated, it is likely that this would generate a more ordered lamellar phase; however, it is less ordered than the branched C_2_C_6_-PBDT derivative. This lower crystallinity of the linear chain derivative is surprising, considering it is commonly used in conjugated polymer design to impart higher crystallinity relative to branched chains and serves to highlight the difficulty in predicting changes in the solid-state packing of conjugated materials.

**Figure 2:**
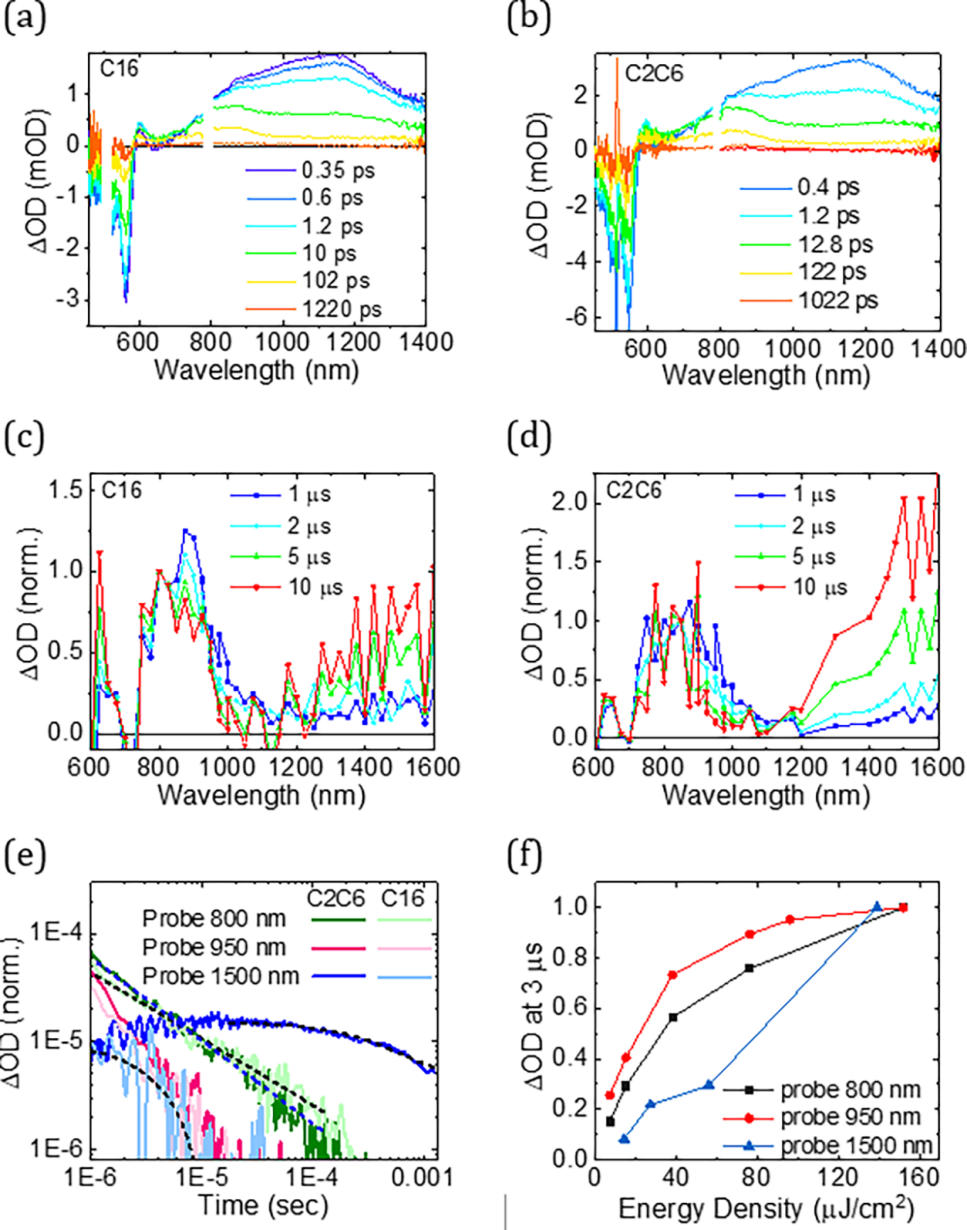
Picosecond transient absorption spectra of thin films of (a) C_16_ and (b) C_2_C_6_ recorded using a 525 nm excitation pulse in the 525–1400 nm spectral range and 5.5 and 11.1 μJ/cm^2^, respectively. Normalized to one, μs transient absorption spectra of (c) C_16_ and (d) C_2_C_6_ film at 1 μs (blue), 2 μs (light blue), 5 μs (green), and 10μs (red). (e) Transient decays of C_2_C_6_ (darker lines) and C_16_ (lighter color lines) films probed at 800 (green), 950 (red), and 1500 nm (blue). Fits with a power law and monoexponential function are in black dashed lines. Lifetimes for C_16_ and C_2_C_6_ triplets are 5 and 450 μs, respectively. (f) Normalized C_16_ excitation dependence of the TA intensity at 3 μs probed at 800 (black), 950 (red), and 1500 (blue) nm. Microsecond TAS spectra and decays were obtained exciting at 520 nm, with an excitation density of 40 μJ/cm^2^. Decays were obtained using band-pass filters.

TA spectroscopy was used to investigate the excited-state dynamics of the thin films of the pristine polymers C_2_C_6_ and C_16_. Figure 2a,b presents their picosecond–nanosecond TA spectra. Both polymer films have similar spectral signatures, which include a bleach band at 560 nm and two positive excited-state absorption bands at ∼800 and ∼1200 nm. The bleach signal matches the steady-state absorption spectra in Figure 1b. The 1200 nm positive band is assigned to polymer singlet excited-state absorption (S_1_) because of its formation with the excitation pulse and picosecond lifetime, as estimated by the global analysis fits in Figure S1. The excited-state absorption at 800 nm takes place directly from S_1_, and according to the global analysis, it has exactly the same yield of ∼55% in both polymer films. It then decays with two time constants, 47 ps and 1.7 ns for C_16_ and 101 ps and 4.8 ns for C_2_C_6_. The 800 nm absorption band is the only excited-state absorption resolved on the nanosecond timescale. Therefore, it is estimated from the bleach amplitude at 1 ns that the yield of long-lived excited states is noteworthy in both polymers (C_2_C_6_ = 9%, C_16_ = 4%) and significantly larger in the more crystalline film structure of C_2_C_6_.

To investigate the long-lived excited states, we conducted μs TA spectroscopy. Figure 2c shows the TA spectrum of the C_16_ film recorded at 1–10 μs and normalized at 800 nm. The spectrum at 1 μs is consistent with the spectrum at 1 ns from Figure 2a. Three different absorption bands can be resolved in the μs TA spectra of C_16_: an 800 nm band, another at 880 nm that decays faster, and a band that absorbs from 1150 to 1700 nm. The kinetics of these different spectral features were studied in Figure 2e. The 800 nm band decays following a power law with an α of 0.7. The power law decay is usually associated with trapped polarons, where the α value indicates the depth of these trap states (the lower the α value, the deeper the trap state).^29,30^ We also conducted experiments in oxygen and nitrogen environments (Figure S2), which did not show any quenching by oxygen, which is consistent with the assignment of the 800 nm signal to polarons. On the other hand, the band that absorbs in the near-infrared, >1200 nm, decays following a monoexponential function, which is associated with first-order processes such as triplet relaxation to the ground state. The 880 nm feature (probed at 950 nm to minimize contamination from the 800 nm band) is not distinctly either power law or monoexponential, and thus it is difficult to make an initial assignment. This ambiguity is exacerbated by the 880 nm feature’s proximity to the 800 nm polaron. The oxygen dependence experiments (Figure S2) showed reversible quenching by oxygen in the TA decay probed at 1500 nm. Triplets react reversibly with oxygen if the triplet energy level is higher than the 0.98 eV threshold, which corresponds to the oxygen singlet energy level. Therefore, the oxygen sensitivity experiment confirms the assignment of the 1500 nm features to polymer triplets. However, the 880 nm feature displays a partial oxygen sensitivity. As such, it is not clear from these experiments whether the feature seen at 880 nm is a triplet or polaron feature.

The assignment of the 800 nm band to polarons and the 1500 nm band to triplets was verified by examining the excitation density dependence of the TA signal amplitude (Figures 2f and S3). The 800 nm band displayed the saturation typical of a second-order process such as bimolecular recombination, while the 1500 nm band amplitude showed a linear dependence typical of a first-order process. As such, these observations are consistent with the assignments made above. The triplet lifetime of the C_16_ polymer is 5 μs (Figure 2e), in agreement with what would be expected for a conjugated polymer with an optical gap of ca. 2 eV from a previous study.^8^ The 880 nm band (probed at 950 nm) also showed a saturation, but its proximity to the 800 nm band again complicates a clear assignment. However, it is clear that the 880 nm region saturates more rapidly than the 800 nm band, which may suggest bimolecular recombination of a charged species.

Next, the branched alkyl-chained polymer, C_2_C_6_, was studied (Figure 2d). Similarly to C_16_, the C_2_C_6_ polymer shows features at 800 and 1500 nm, assigned to polarons and triplets, respectively. However, the decay kinetics of the C_2_C_6_ polymer triplet (Figure 2e) are distinctly different from the ones obtained for the C_16_ polymer. There is a rise in the C_2_C_6_ triplet from 1 to 10 μs, reaching a plateau at ∼20 μs, then finally decaying monoexponentially with a notably long lifetime of 450 μs. This is 2 orders of magnitude longer than the triplet lifetime of C_16_ and 2–3 orders of magnitude longer than the triplets of well-known polymers with a similar bandgap such as PCDTBT, APFO3, and IF8TBTT, and over 4 times longer than the low-triplet-energy small-molecule rubrene.^11,31^Table S1 compares the triplet lifetime obtained in this work with small molecules and polymers with different bandgaps seen in the literature.^8,11,24,31−48^ The rise in triplet population on the μs timescale for C_2_C_6_ indicates that these triplets are not formed from standard intersystem crossing. Instead, they are formed upon a bimolecular charge recombination process, as can be observed by the C_2_C_6_ bimolecular polaron decay kinetics matching the rise of the triplets (Figure S4). As a comparison, C_16_ triplets are already formed by 1 μs. From the normalized early nanosecond TA spectra of C_16_ and C_2_C_6_, in Figure S5, there is no evidence of the polymer triplet peak (1200 nm), which shows that the triplets in C_16_ are also most likely formed via bimolecular polaron recombination, although this clearly happens at a much faster rate than in C_2_C_6_.

**Figure 3:**
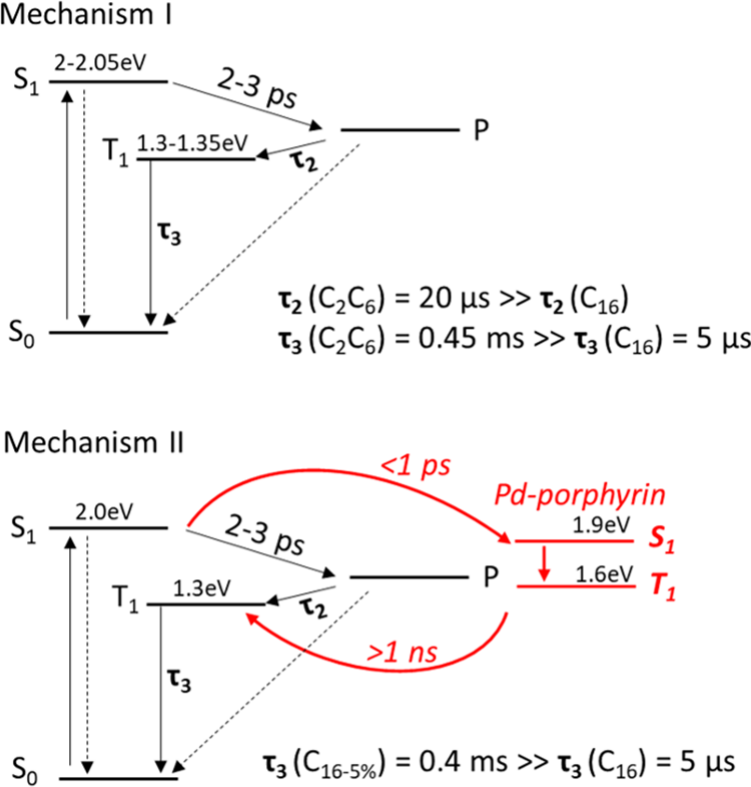
State diagram depicting the two mechanisms of triplet generation and their time constants resolved in this study. Mechanism I corresponds to polaron-mediated triplet generation, while mechanism II corresponds to Pd porphyrin-mediated triplet generation. S_1_, first singlet excited state, T_1_, first triplet excited state, and P, first polaron state. S_1_ and T_1_ energies are presented according to experimental absorption and phosphorescence spectroscopy data, as discussed in the text. The black straight lines and arrows represent the excited-state processes in the polymer, and the red curved lines represent the processes involving Pd porphyrin.

In Figure 3, Mechanism I, we summarize the processes resolved in the excited-state dynamics of the C_16_ and C_2_C_6_ films. Excitation to the polymer S_1_ state is followed by competing exciton relaxation to the ground state and dissociation to polarons. From the bleach recovery, we estimate an upper limit of the polaron generation yield of 55%, which is high for such pristine polymers.^49,50^ A proportion of the polarons recombine quickly on the tens of picosecond timescale to the ground state via a geminate recombination process,^51,52^ leaving about 4% in C_16_ and 9% in C_2_C_6_ of the total photoexcitation as long-lived polarons, which shows a doubling of the yields in the more crystalline polymer film. The triplet states in both polymers are the product of bimolecular recombination of the long-lived polarons. It has often been observed that changes in crystallinity can lead to changes in bimolecular recombination rates.^53^ Here, significantly slower triplet generation is found in the more crystalline C_2_C_6_, which can be linked to a larger energy barrier for recombination of charges due to the formation of low-energy crystalline regions in the film.^54^ Furthermore, the lifetime of the polymer triplets of C_2_C_6_ is close to 2 orders of magnitude longer than C_16_ triplets, which can be linked to C_2_C_6_’s higher crystallinity and closer π–π stacking distance. These structural features can slow down intersystem crossing via possibly larger steric constraints and limited out-of-plane vibrations, which are known to reduce spin–orbit coupling^55^ and via limited triplet diffusion after population of the lowest-lying triplets. This is a remarkable difference in the triplet lifetime for two polymers with an identical conjugated backbone.

### Triplet Sensitization Mechanism

It was discussed in the Introduction Section that sensitization with porphyrins is a strategy to increase the yield and lifetime of triplets. We incorporated 5 mol % of Pd porphyrin in the C_16_ structure to investigate the changes in dynamics caused by the high intersystem crossing yields in metal porphyrins. Here, we calculated the mol % of the Pd porphyrin from the co-monomer feed ratio (see the synthesis section in the SI for clarification notes). The mol % of Pd porphyrin was chosen based on Andernach et al., who observed a high triplet yield for 5 mol % Zn porphyrin loading in a poly(phenyl-bithiophene) polymer.^11^ Pd was chosen to target higher spin–orbit coupling with the heavier atom, while the metalation of tetrabenzoporphyrins with Pd is much more synthetically facile than Pt.

The Soret and Q bands of the incorporated porphyrin can be seen in the UV–vis spectrum shown in Figure 1b. From the onset of the UV–vis spectrum, the S_1_ energy of the polymer backbone in film is estimated at 2.05 and 2.0 eV for C_2_C_6_ and C_16_ (C_16_-5%), respectively. Assuming a typical singlet–triplet energy gap for conjugated polymers (0.7 eV), the polymers triplet should reside around 1.35 and 1.3 eV, respectively.^28^ Phosphorescence measurements of the Pd porphyrin solutions showed its triplet to be at 1.6 eV (Figure S6). Time-dependent density functional theory (TD-DFT) calculations (B3LYP/6-31G*—LANL2DZ pseudopotential for the Pd atom) on the monomer and several oligomers were carried out upon polymer design to assess the polymer S_1_ singlet and T_1_ triplet excited states relative to Pd porphyrin. These were found to change with oligomer length but generally lay close to the Pd porphyrin states (Figure S7), especially for the trimer and tetramer for which the polymer triplet falls below Pd porphyrin to enable triplet energy transfer. The calculations agree with the experimental data received from Figure 1b and our estimates of the polymer and Pd porphyrin triplets are summarized in Figure 3. From our experimental spectroscopy data, we do not observe evidence for changes in S_1_ and T_1_ of the polymer or Pd porphyrin upon copolymerization (Figure S8).

**Figure 4:**
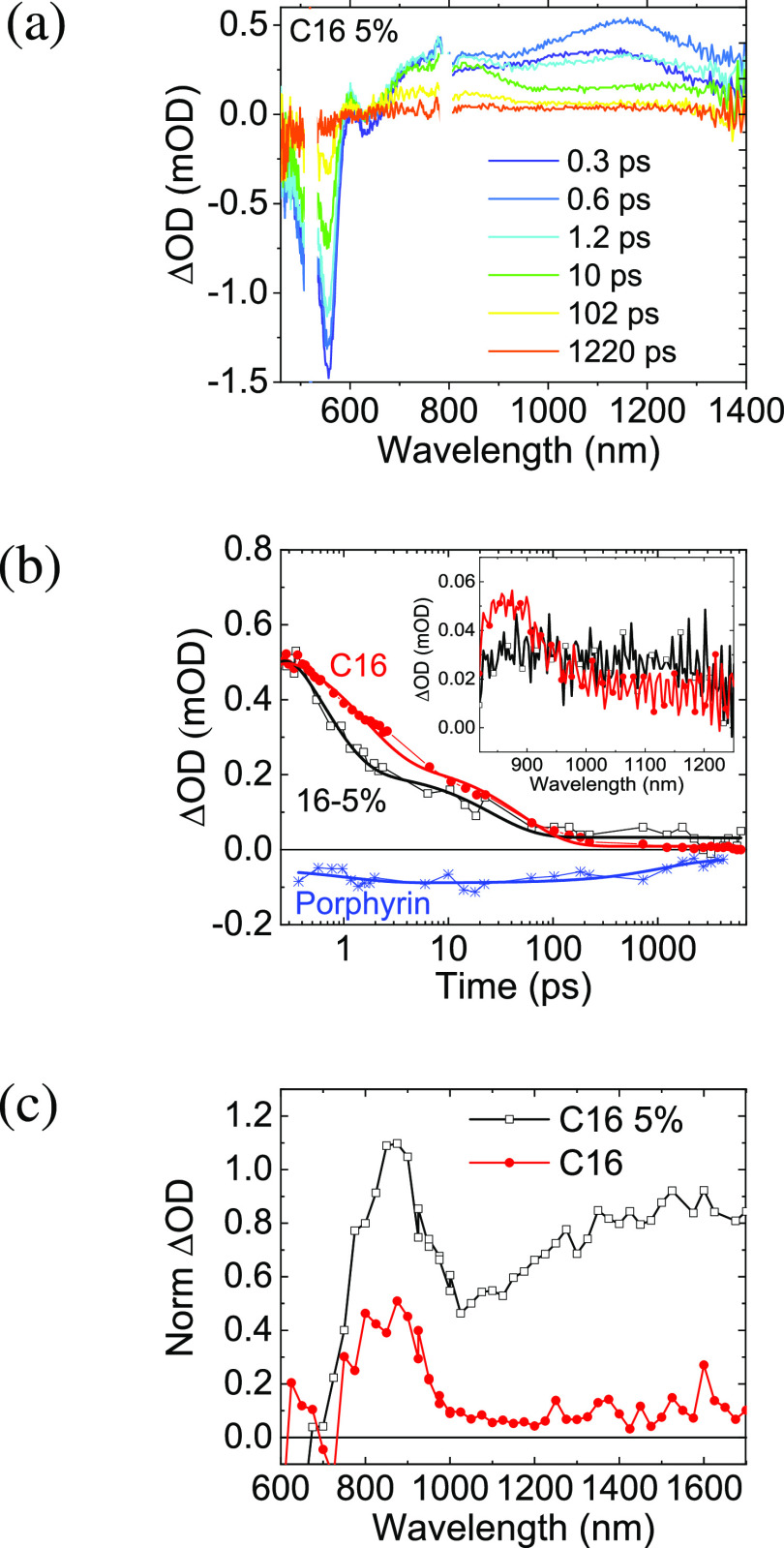
Transient absorption spectra of thin films of (a) C_16_-5%, recorded using a 525 nm excitation pulse. (b) Decay of S_1_ excited-state absorption in C_16_ and C_16_-5% (red and black, respectively), probed at 1270 nm, and the Pd porphyrin bleach dynamics in C_16_-5% (blue). The inset presents the average transient absorption spectrum at 2–4.7 ns in the near-infrared identifying excited-state population differences between C_16_ (red) and C_16_-5% (black). (c) Transient absorption spectra at 1 μs normalized per photon absorbed of C_16_ (red, circles) and C_16_-5% (black, open squares), obtained with 520 nm excitation with an excitation density of 40 μJ/cm^2^. For the decays, band-pass filters were used to avoid the white light effect.

The C_16_-5% TA spectra are presented in Figure 4a. The pump pulse at 525 nm solely excites the polymer. The spectra are similar to that of the pristine C_16_ polymer but have an additional Pd porphyrin bleach at 625 nm formed on the picosecond timescale. The amplitude of the Pd porphyrin bleach was extracted by deconvoluting this signal from the rest of the spectra. Figure 4b presents the kinetics of the polymer S_1_ exciton in C_16_ and C_16_-5% and the bleach of the porphyrin in C_16_-5%. It reveals strong quenching of the polymer S_1_ lifetime in the presence of Pd porphyrin and a picosecond rise time of the Pd porphyrin bleach. Global analysis of the TA spectra of C_16_-5% (Figure S1 and Table S2) quantifies the quenching of polymer S_1_ to be 49%, with a time constant of 0.97 ps. This S_1_ decay lifetime matches the picosecond rise time of the porphyrin bleach and reveals that Pd porphyrin is populated from the S_1_ state of the polymer. The fluorescence spectra of C_16_ and C_16_-5% (Figure S9) also indicate strong quenching of S_1_ in C_16_-5%, but with a 76% quenching yield, which is higher than that estimated by TA. This discrepancy is likely to be due to a process faster than the instrument response function of our TA spectrometer. According to previous reports, the mechanism of population of the porphyrin excited state is assigned to Förster resonance energy transfer (FRET) from the polymer S_1_.^11^ Electron transfer is another possible mechanism for Pd porphyrin excited states population, according to the highest occupied molecular orbital–least unoccupied molecular orbital (HOMO–LUMO) energy alignment in Table S4 (obtained from cyclic voltammetry values from literature),^56,57^ but in that case, an increase in the polymer polaron signal would be expected on the picosecond timescale. Instead, we observed a 55 ± 5% drop in polaron signal in C_16_-5% compared to C_16_ (as estimated at 100 ps in Figure S10 and Table S3), matching the singlet quenching yields and ruling out the charge-transfer process as an explanation.

Figure 4c shows that the polymer triplet intensity increases by a factor of close to 7.9 times with the introduction of Pd porphyrin in the polymer structure; this cannot be assigned to absorption by Pd porphyrin triplets, which is known to appear at ∼700 nm.^58^ Therefore, we link the formation of a higher population of polymer triplets to a back energy transfer of triplets from Pd porphyrin to the polymer. The triplets of C_16_ formed via energy transfer from and to Pd porphyrin show a lifetime exceeding that of the pristine polymer triplet by 2 orders of magnitude (from 5 to 400 μs, Figure S11). Interestingly, the lifetime of C_16_-5% triplets is of the same order as the C_2_C_6_ triplets. The triplet excited-state dynamics in the C_16_-5% is summarized in Figure 3.

## Conclusions

In this study, we report two polymers based on the BDT-T monomer with triplet states with a nearly millisecond lifetime, which are some of the longest triplet lifetimes reported to date for red-emitting conjugated polymer films, closing the gap with blue absorbing molecular crystals.^32−34,38,43^ This is achieved using two different triplet generation mechanisms. The first is by generating triplets via slow bimolecular electron–hole recombination, and the second by incorporating a Pd porphyrin into the polymer backbone to act as a triplet sensitizer, whereby polymer triplets are formed by back energy transfer from the porphyrin.

By connecting two different alkyl side chains to the BDT-T core, two different polymer film morphologies are created: the more amorphous linear C_16_ vs the more crystalline branched C_2_C_6_. Transient absorption spectroscopy experiments show that bimolecular recombination governs triplet generation in both polymers, but generation is slowed down to tens of microseconds in the more crystalline C_2_C_6_ polymer film. Furthermore, the more crystalline nature of the C_2_C_6_ film enables a substantially longer triplet lifetime compared to the amorphous C_16_, by nearly 2 orders of magnitude to the impressive value of nearly half a millisecond. This result highlights that the triplet dynamics of conjugated polymers can be sensitive to even subtle changes in chemical structure.

In the second part of this study, we covalently incorporate a Pd porphyrin at 5 mol %, which results in an 8-fold increase in the yields of polymer triplets by utilizing the high intersystem crossing yield of Pd porphyrin, and substantially increases the polymer triplet lifetime to 0.4 ms. This study therefore showcases successful strategies to enhance triplet populations and lifetimes vital for pushing optical bandgaps further into the red and near-infrared without compromising excited-state lifetimes, which is crucial not only for organic electronics but also for sensors, communications, and bioapplications such as photodynamic therapy and drug delivery via photocaging.

## Supporting Information Available

Synthesis and methods and additional spectroscopic characterization (PDF)
