# Supplementary material for: Generating Long-Lived Triplet Excited States in Narrow Bandgap Conjugated Polymers
Source: J Am Chem Soc. 2023 Feb 3;145(6):3507–14. doi: 10.1021/jacs.2c12008 (PMC9936540; doi:10.1021/jacs.2c12008)
Supplement: Supplementary file 1 — ja2c12008_si_001.pdf [file ja2c12008_si_001.pdf]

# Supporting Information

**Generating long-lived triplet excited states in narrow bandgap conjugated polymers**

Jose M. Marin-Beloqui[1,2†‡], Daniel G. Congrave[3‡], Daniel T. W. Toolan[4], Stephanie Montanaro[5], Junjun Guo[1], Iain A. Wright[5,6], Tracey M. Clarke[1*], Hugo Bronstein[3*], Stoichko D. Dimitrov[7*]

1 - Department of Chemistry, University College London, London WC1H 0AJ, UK.

2 - Department of Physical-Chemistry, University of Málaga, Campus de Teatinos, Málaga, 29071 Málaga, Spain

3 - Department of Chemistry, University of Cambridge, Cambridge, CB2 1EW, UK.

4 - Department of Chemistry, Dainton Building, The University of Sheffield, Brook Hill, Sheffield S3 7HF, UK.

5 - Department of Chemistry, Loughborough University, Loughborough, LE11 3TU, UK.

6 - School of Chemistry, University of Edinburgh, Edinburgh, EH9 3FJ, UK.

7 - Department of Chemistry, Queen Mary University of London, London E1 4NS, UK.

## General synthesis

$^{1}$H NMR spectra were recorded on a 400 MHz Avance III HD Spectrometer, 400 MHz Smart Probe Spectrometer or a 500 MHz DCH Cryoprobe Spectrometer in the stated solvent using residual protic solvent as the internal standard. $^{1}$H NMR chemical shifts are reported to the nearest 0.01 ppm. The coupling constants (*J*) are measured in Hertz. $^{13}$C NMR spectra were recorded on the 500 MHz DCH Cryoprobe Spectrometer in the stated solvent using the residual protic solvent as the internal standard. $^{13}$C NMR chemical shifts are reported to the nearest 0.1 ppm. Mass spectra were obtained using a Waters LCT, Finnigan MAT 900XP or Waters MALDI micro MX spectrometer at the Department of Chemistry, University of Cambridge. Thin layer chromatography (TLC) was carried out on silica gel and visualized using UV light (254, 365 nm). Flash chromatography was carried out on a Biotage® Isolera automated flash chromatography machine on 60 micron silica gel cartridges purchased from Biotage®. Number-average ($M_n$) and weight-average ($M_w$) molecular weights were determined against a polystyrene standard using an Agilent Technologies 1200 series GPC in chlorobenzene at 80 °C. All commercial chemicals were of ≥95% purity and were used as received without further purification. Anhydrous solvents were purchased from Sigma Aldrich or Acros Organics and used as received

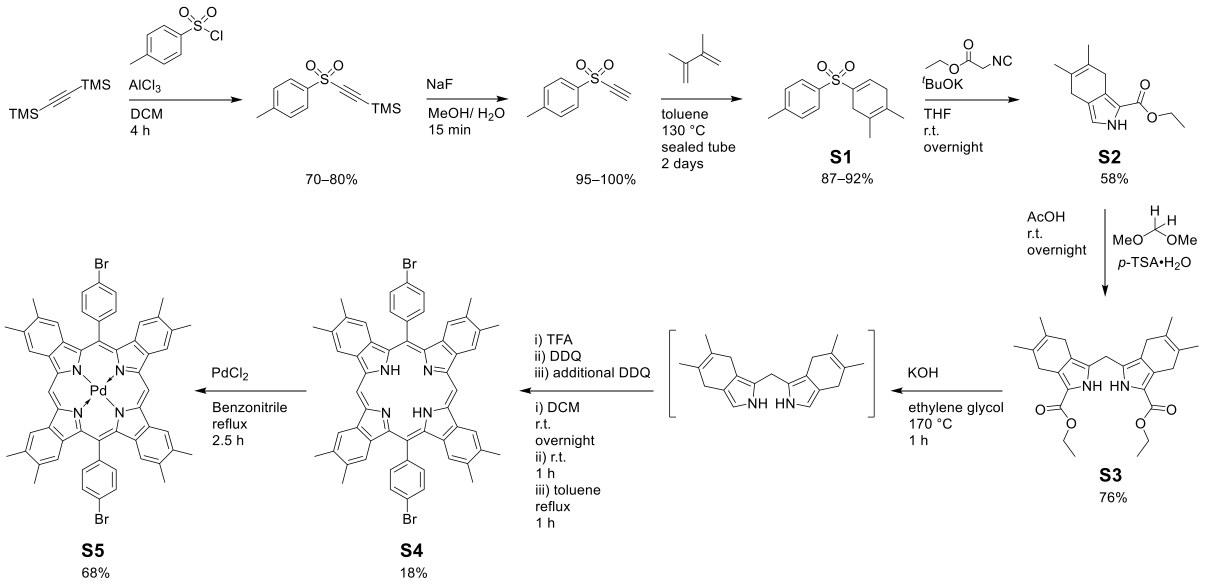


Trimethyl(tosylethynyl)silane[1] and Tosylethyne[2] were synthesised according to literature procedures.

**1-Tosyl(4,5-dimethylcyclohexa-1,4-diene)** (**S1**). A solution of tosylethyne (13.2 g, 73.2 mmol, 1.00 eq.) and 2,3-dimethylbutadiene (7.26 g, 88.4 mmol, 1.20 eq.) in dry toluene (75 mL) was degassed for 20 min. The mixture was then distributed amongst Biotage™ 20 mL microwave vials or Ace™ pressure tubes (Sigma Aldrich) (half filling them), which were sealed under argon and heated in a 130 °C preheated oil bath for 40 h. The reaction mixtures were recombined and evaporated under reduced pressure. The residue was purified by flash chromatography on silica gel (eluent: chloroform) to obtain 1-tosyl(4,5-dimethylcyclohexa-1,4-diene) (**S1**) as a white powder (16.8–17.7 g, 64.0–67.5 mmol, 87–92%). $^1$H NMR data were in accordance with those reported in the literature.[3] $^1$H NMR (400 MHz, $CDCl_3$) δ (ppm) = 7.78 (d, *J* = 8.3 Hz, 2H), 7.34 (d, *J* = 8.3 Hz, 2H), 7.02 – 6.98 (m, 1H), 2.86 (dd, *J* = 5.8, 4.0 Hz, 2H), 2.73 (t, *J* = 7.7 Hz, 2H), 2.45 (s, 3H), 1.63 (s, 6H).

**2-Ethylcarbonyl-4-7-dihydro-5,6-dimethyl-2*H*-isoindole** (**S2**). This compound was prepared according to a modified literature procedure for an analogue.[4] *t*-BuOK (4.96 g, 44.2 mmol, 1.05 eq.) was added to dry, degassed THF (38 mL) under argon and the resulting suspension was cooled to 0 °C in an ice bath (*note: a large stirrer bar is recommended*). A degassed solution of ethyl isocyanoacetate (5.00 g, 44.2 mmol, 1.05 eq.) in dry THF (19 mL) was then added dropwise, yielding an orange-yellow milky suspension. The reaction mixture was stirred at 0 °C for 30 min, and then a solution of 1-tosyl(4,5-dimethylcyclohexa-1,4-diene) (**S1**) (11.05 g, 42.0 mmol, 1.00 eq.) in dry, degassed THF (38 mL) was added dropwise. The resulting thick yellow suspension was warmed to room temperature and stirred overnight. The solvent was removed under reduced pressure and DCM (200 mL) and water (200 mL) were added to the residue. The layers were separated and the aqueous layer was further extracted with DCM (2 × 200 mL). The organic layers were combined, and stirred with activated charcoal and $Na_2SO_4$. After *ca.* 15 min, the mixture was filtered through celite and the solvent removed under reduced pressure. The residue was recrystallized by refluxing the material in *n*-hexane (200 mL) and slowly adding toluene until full dissolution was observed. A tan powder fell from the solution after cooling the mixture to room temperature for 3 h, and then in a −20 °C freezer for 2 h. The precipitate was isolated via filtration and washed with −78 °C pentane (2 × 25 mL) to obtain 2-ethylcarbonyl-4-7-dihydro-5,6-dimethyl-2*H*-isoindole (**S2**) as a tan powder (5.66 g, 25.8 mmol, 58%). $^1$H NMR data were in accordance with those reported in the literature.[3] $^1$H NMR (400 MHz, $CDCl_3$) δ (ppm) = 8.86 (bs, 1H),

6.71 (d, $J$ = 2.8 Hz, 1H), 4.34 (q, $J$ = 7.1 Hz, 2H), 3.38 (bs, 2H), 3.15 (bs, 2H), 1.82 (s, 3H), 1.79 (s, 3H), 1.39 (t, $J$ = 7.1 Hz, 3H).

**Bis(3-ethoxycarbonyl-5,6-dimethyl-4,7-dihydro-2*H*-isoindolyl)methane** (**S3**). This compound was prepared according to a modified literature procedure for an analogue.[5] A 2-ethylcarbonyl-4-7-dihydro-5,6-dimethyl-2*H*-isoindole (**S2**) (5.66 g, 25.8 mmol, 2.00 eq.), dimethoxymethane (0.98 g, 12.9 mmol, 1.00 eq.) and *para*-toluenesulfonic acid (245 mg, 1.29 mmol, 0.10 eq.) were added to acetic acid (130 mL) and the resulting mixture was stirred at room temperature for 24 h under air. The reaction mixture was then poured into ice water (800 mL). A light yellow precipitate formed, which was isolated by filtration and washed copiously with water. The filtrand was refluxed in ethanol (300 mL) for 4 h. The insoluble material was then isolated via filtration (without cooling) and washed with further room temperature ethanol (3 × 20 mL) to obtain bis(3-ethoxycarbonyl-5,6-dimethyl-4,7-dihydro-2*H*-isoindolyl)methane (**S3**) as a white powder (4.41 g, 9.79 mmol, 76%). $^1$H NMR (400 MHz, DMSO) δ (ppm) = 11.29 (bs, 2H), 4.21 (bs, 4H), 3.79 (bs, 2H), 3.19 (bs, 4H), 2.87 (bs, 4H), 1.69 (bs, 12H), 1.27 (bs, 6H); HRMS (ESI): *m/z* 450.2478 [M$^+$]. Calcd. for $C_{27}H_{34}N_2O_4^+$: 450.2513.

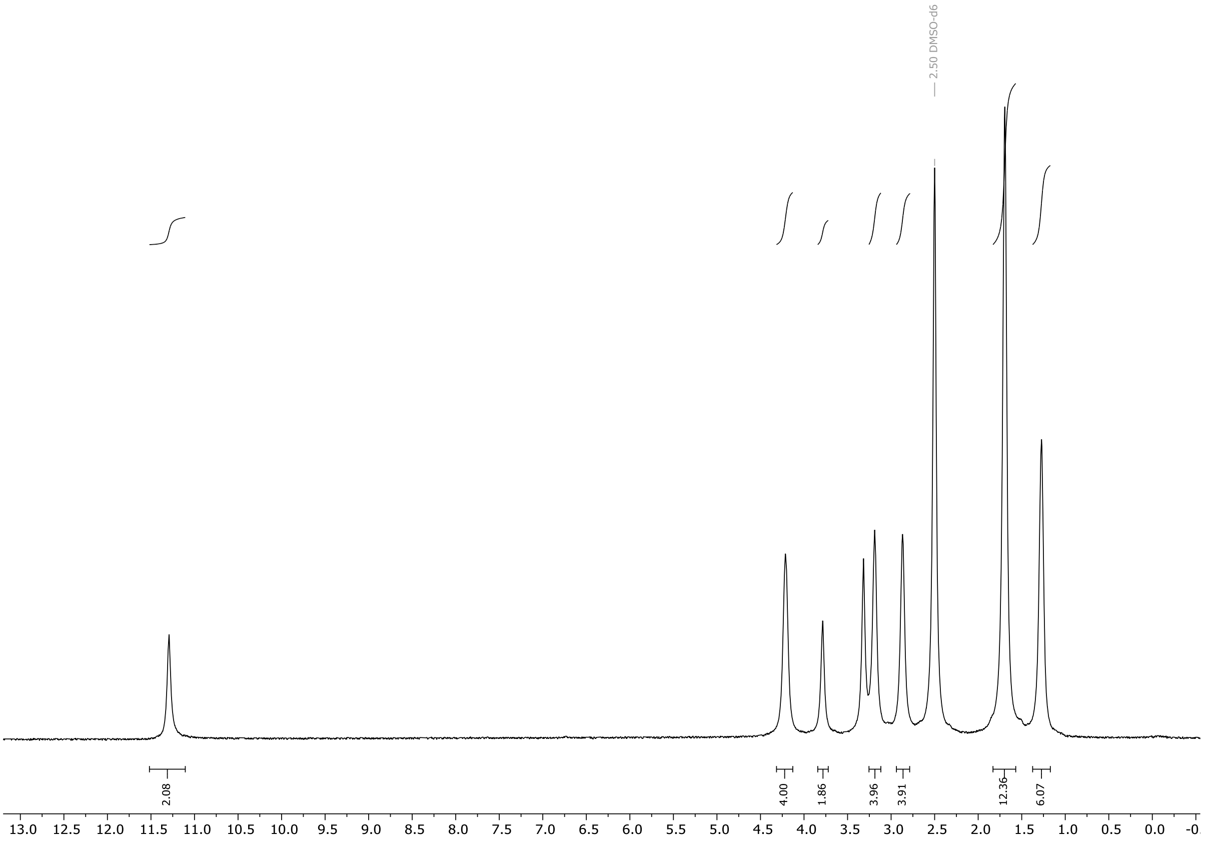


**NMR spectrum 1.** $^1$H NMR spectrum of **S3**.

**5,15-Di(4-bromophenyl)-$2^2,2^3,7^2,7^3,12^2,12^3,17^2,17^3$-octamethyltetrabenzoporphyrin** (**S4**). This compound was prepared according to a modified literature procedure for an analogue.[6] A suspension of bis(3-ethoxycarbonyl-5,6-dimethyl-4,7-dihydro-2*H*-isoindolyl)methane (**S3**) (1.49 g, 3.31 mmol, 1.00 eq.) and KOH (1.85 g, 16.8 mmol, 5.06 eq.) in ethylene glycol was thoroughly sparged with argon for 30 min. The reaction flask was then immersed in a preheated 170 °C oil bath and stirred under argon for *ca.* 1 h until no precipitate remained, resulting in a dark orange solution *(note: periodic swirling of the reaction flask helps to remove any* **S3** *that gets stuck to the flask walls*). The reaction flask was immediately cooled in an ice bath and the reaction mixture was poured into DCM (300 mL). Water (200 mL) was added and the layers were separated. The aqueous layer was further extracted with DCM (3 × 50 mL) and the combined organic layers were subsequently washed with water (2 × 100 mL), brine (2 × 100 mL) and further water (1 × 100 mL), dried over $Na_2SO_4$ and the solvent evaporated under reduced pressure. The resulting dark brown solid was dried under high vacuum to a consistent weight to give the presumed intermediate bis(dimethyl-4,7-dihydro-2*H*-isoindolyl)methane (920 mg, 3.00 mmol, 90%) which was used immediately without further purification. Bis(dimethyl-4,7-dihydro-2*H*-isoindolyl)methane (920 mg, 3.00 mmol, 1.00 eq.) and 4-bromobenzaldehyde (555 mg, 3.00 mmol, 1.00 eq.) were combined in dry, degassed DCM (400 mL) and stirred protected from light under argon. Trifluoroacetic acid was immediately added (65 mg, 0.6 mmol, 0.05 eq.) and the mixture was stirred at room temperature overnight. DDQ (1.02 g, 4.50 mmol, 1.50 eq.) was added and the reaction stirred at room temperature for 1 h affording a dark purple mixture. The solvent was evaporated under reduced pressure and the residue was redissolved in degassed toluene (500 mL) with DDQ (1.36 g, 6.00 mmol, 2.00 eq.) and refluxed under argon for 1 h to give a dark green mixture. The solvent was again removed under reduced pressure. The residue was then purified by dissolving it in DCM (*ca.* 250 mL) and passing it through a short pad of silica (⌀ 10 cm × 1 cm) eluting firstly with DCM (*ca.* 5 L) and then boiling $CHCl_3$. Dark green fractions were collected until brown baseline impurities began to elute, and were then combined. The solvent was evaporated under reduced pressure and the residue was suspended in hexane (*ca.* 30 mL) and sonicated. The mixture was cooled to *ca.* 0 °C and the solid was isolated via filtration and washed with −78 °C pentane (3 × 3 mL) to obtain 5,15-di(4-bromophenyl)-$2^2,2^3,7^2,7^3,12^2,12^3,17^2,17^3$-octamethyltetrabenzoporphyrin (**S4**) as a dark green powder (250 mg, 0.27 mmol, 18%). NMR data were obtained for the bis(trifluoroacetate) dication salt due to its improved solubility.[5] $^1$H NMR (400 MHz, $CDCl_3$, TFA) δ (ppm) = 10.87 (s, 2H), 9.10 (s, 4H), 8.33 (d, *J* = 8.3 Hz, 4H), 8.21 (d, *J* = 8.3 Hz, 4H), 7.41 (s, 4H), 2.82 (s, 12H), 2.53 (s, 12H), -0.57 (s, $4H_{NH}$); $^{13}$C NMR (126 MHz, $CDCl_3$, TFA) δ (ppm) = 140.9, 140.7, 139.1, 138.5, 137.8, 136.1, 133.0, 131.9, 130.8, 125.3, 125.0, 123.5, 113.8, 91.7, 21.5, 21.1; HRMS (ESI): *m/z* 930.1944 [$M^+$]. Calcd. for $C_{56}H_{44}N_4{}^{79}Br_2{}^+$: 930.1927; UV/ vis ($CH_2Cl_2$) $\lambda_{max}$ B region: 424, 441, Q region: 576, 607, 615, 623, 669.

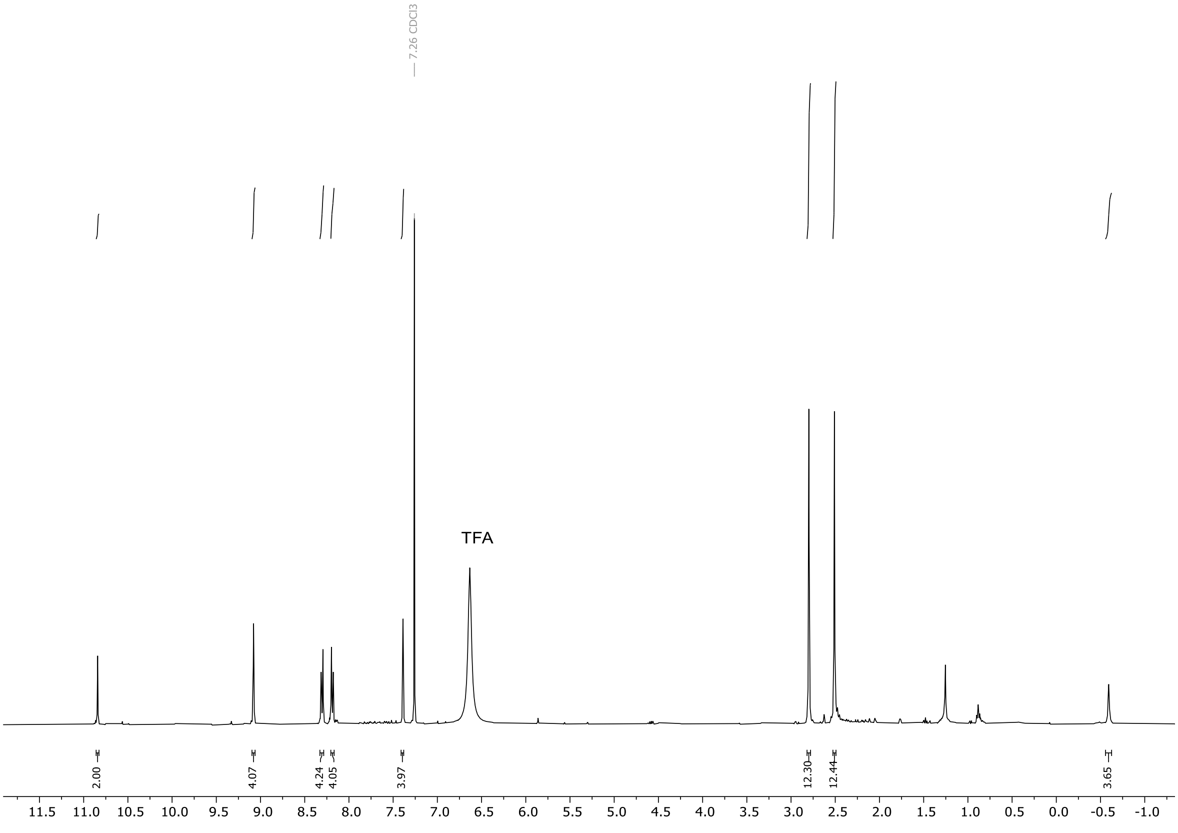


**NMR spectrum 2.** $^{1}H$ NMR spectrum of **S4**.

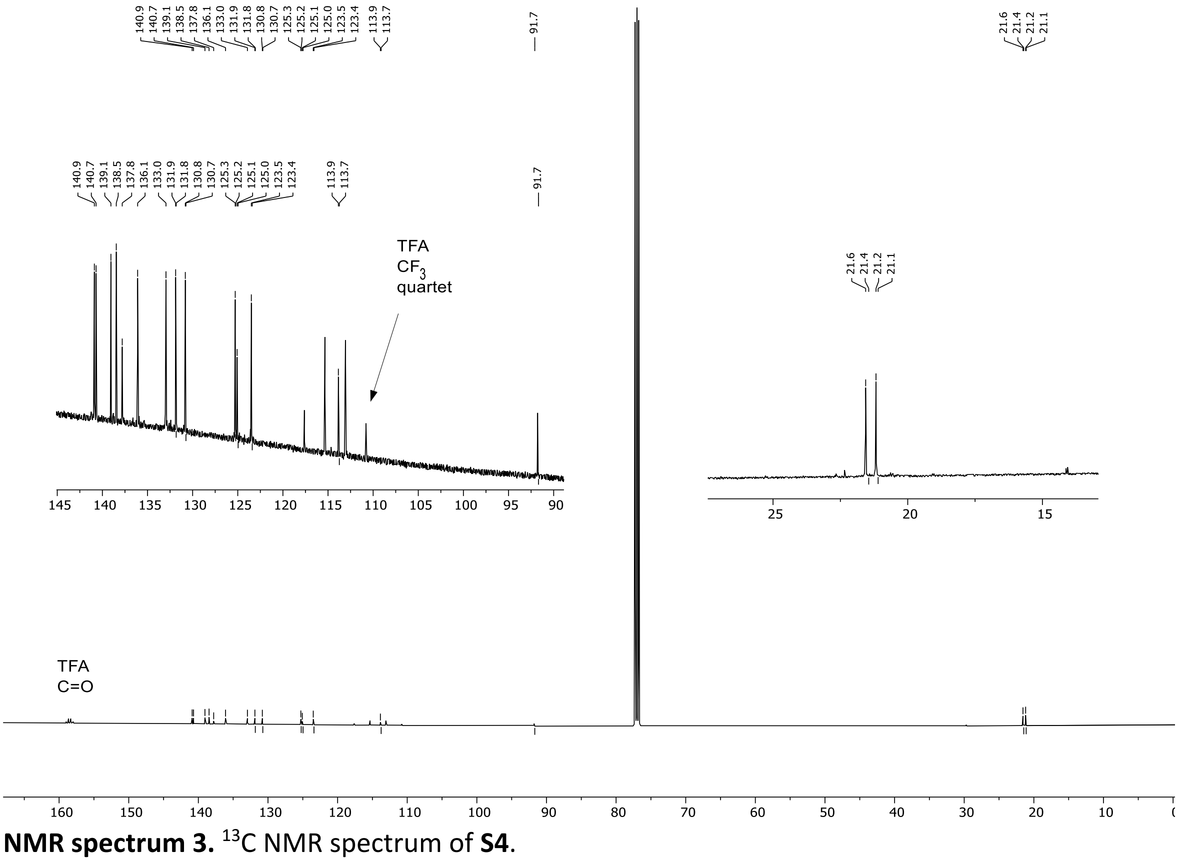


**NMR spectrum 3.** $^{13}C$ NMR spectrum of **S4**.

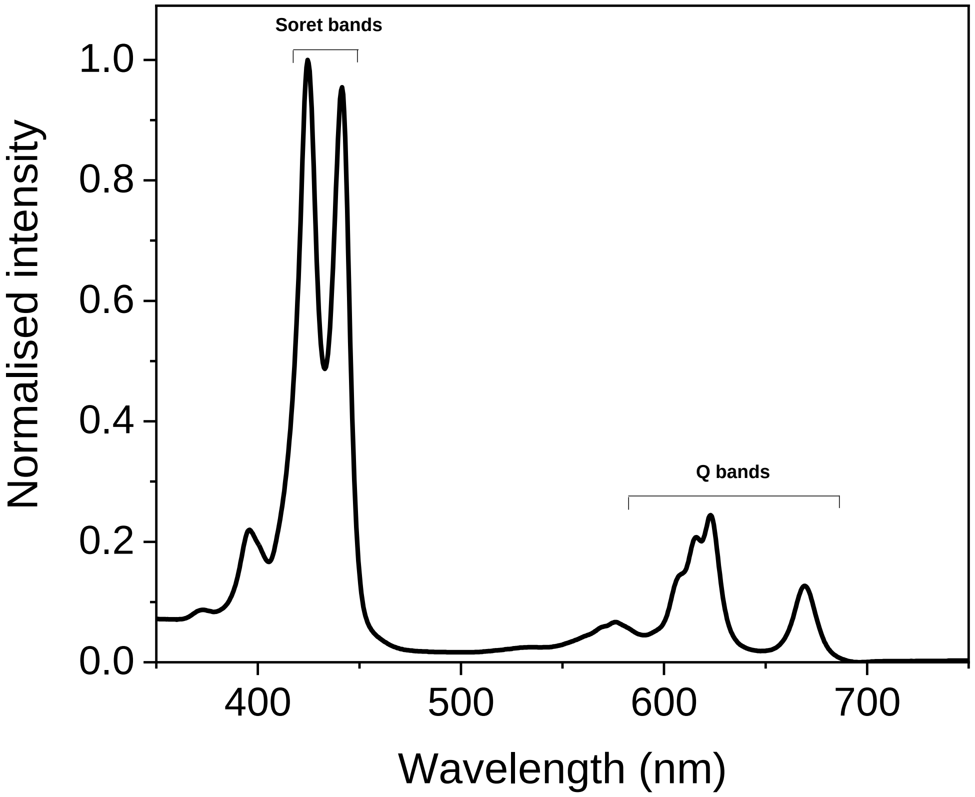


**UV-vis spectrum 1.** Absorption spectrum in $CH_2Cl_2$ of **S4**.

**Palladium-5,15-di(4-bromophenyl)-$2^2,2^3,7^2,7^3,12^2,12^3,17^2,17^3$-octamethyltetrabenzoporphyrin** (**S5**). $PdCl_2$ (63 mg, 0.35 mmol, 3.3 eq.) was added to benzonitrile (50 mL) and the resulting mixture was heated in a 100 °C oil bath for 30 min while sparging with argon. 5,15-Di(4-bromophenyl)-$2^2,2^3,7^2,7^3,12^2,12^3,17^2,17^3$-octamethyltetrabenzoporphyrin (**S4**) (100 mg, 0.11 mmol, 1.00 eq.) was then added and the mixture was vigorously refluxed under argon for 24 h. The reaction was monitored by UV-vis spectroscopy and stopped when the free base (**S4**) could no longer be detected. The solvent was evaporated under reduced pressure and the residue dissolved in DCM (*ca.* 50 mL). The mixture was passed through a pad of celite (Ø 5 cm × 0.5 cm) eluting with 2% vol. MeOH in DCM until the eluent was clear. The solvent was then evaporated under reduced pressure and the residue triturated with pentane (30 mL) and filtered to obtain the crude product as a black powder (107 mg, 0.10 mmol, 96%). It was suspended in methanol (15 mL), refluxed for 6 h, cooled to room temperature and the resulting fine suspension centrifuged. The precipitate was recovered by firstly filtering the solution and then dissolving the material that remained in the centrifuge tube in acetone. The filtered solid and acetone extracts were combined and the solvent evaporated under reduced pressure. The residue was triturated with pentane (30 mL), filtered, and subsequently washed with MeOH (3 × 3 mL) and pentane (3 × 3 mL) to afford palladium-5,15-di(4-bromophenyl)-$2^2,2^3,7^2,7^3,12^2,12^3,17^2,17^3$-octamethyltetrabenzoporphyrin (**S5**) as a dark green solid (75 mg, 0.07 mmol, 68%). While well soluble in aromatic and chlorinated solvents, the complex appears to aggregate at the concentrations required to obtain NMR spectra, resulting in poor signal-to-noise even at elevated temperatures. This has been previously reported for analogues.[5–7] $^1$H NMR (500 MHz, $d_5$-pyridine, 95 °C) δ (ppm) = 10.85 (s, 2H), 9.34 (s, 4H), 8.25 (d, *J* = 7.8 Hz, 4H), 8.04 (d, *J* = 7.7 Hz, 4H), 7.27 (s, 4H), 2.82 (s, 12H), 2.61 (s,

12H); HRMS (ESI): *m/z* 1034.0796 [$M^+$]. Calcd. for $C_{56}H_{42}N_4{}^{79}Br_2{}^{106}Pd^+$: 1034.0805; UV/ vis (DMF) $\lambda_{max}$ 421 (B), 622 (Q).

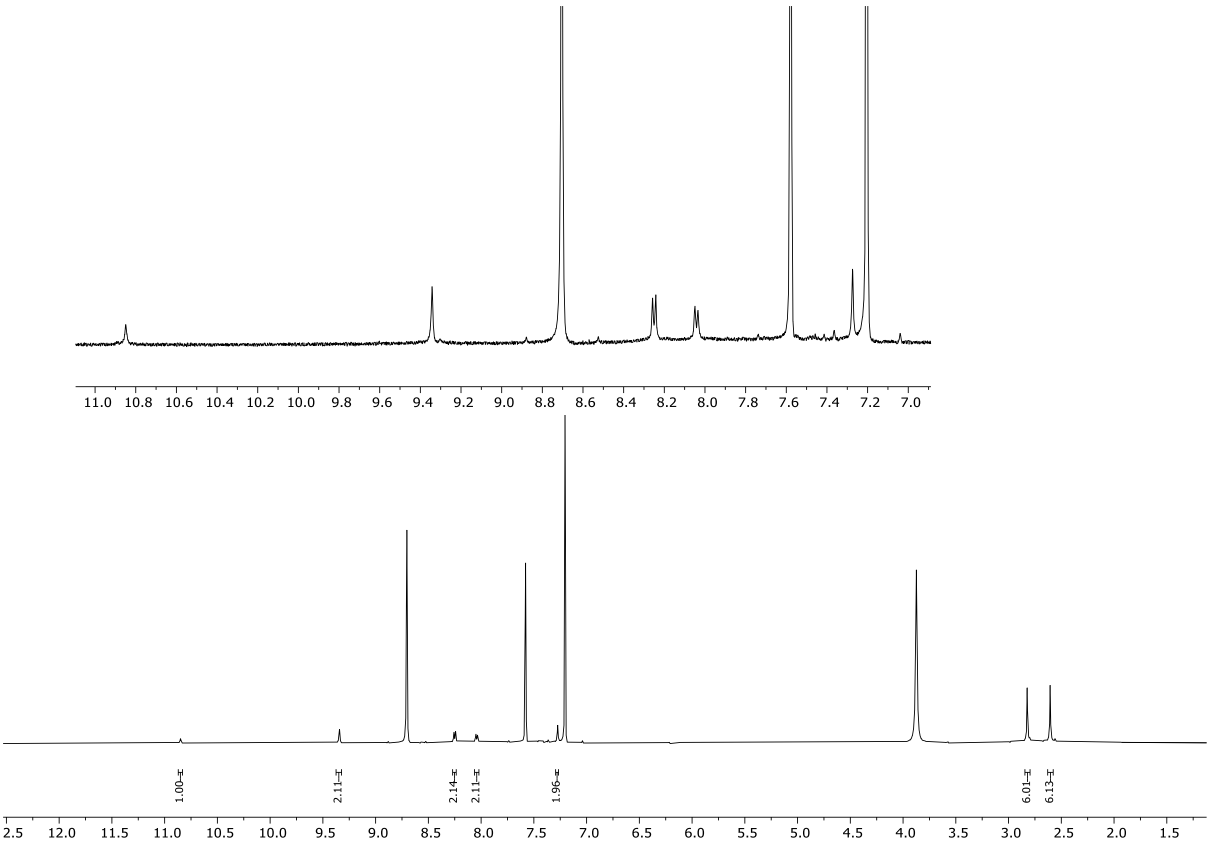


**NMR spectrum 4.** $^1$H NMR spectrum of **S5**.

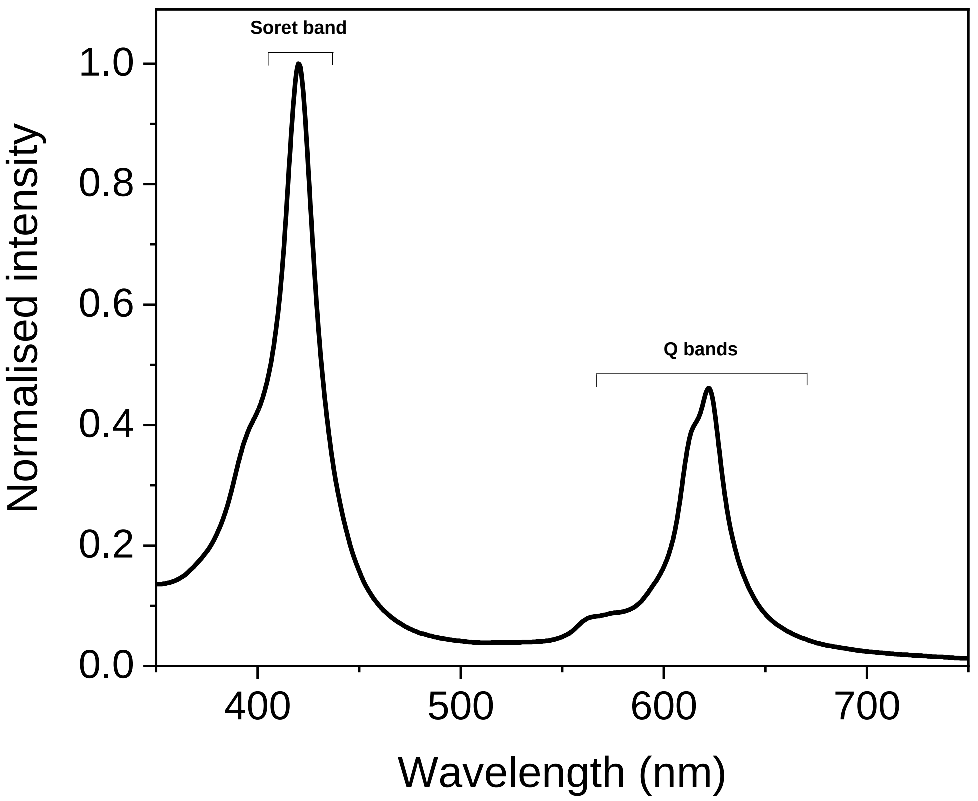


**UV-vis spectrum 2.** Absorption spectrum in DMF of **S5**.

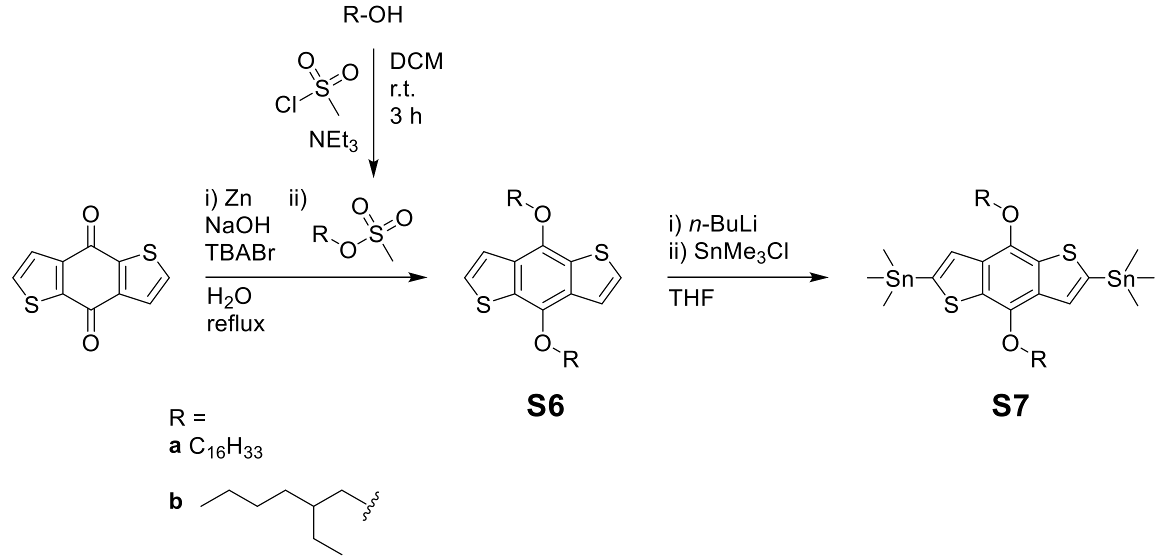


**4,8-Di(hexadecyloxy)benzodithiophene** (**S6a**). Prepared based on modified literature procedures.[8,9]

*Representative preparation of mesylalkane*

1-Hexadecanol (5.02 g, 20.7 mmol, 1.00 eq.) and methanesulfonylchloride (2.61 g, 22.8 mmol, 1.10 eq.) were dissolved in dry DCM (60 mL) under argon to give a colourless solution. Triethylamine (2.30 g, 22.8 mmol, 1.10 eq.) was then added dropwise (*the reaction is exothermic and causes the mixture*

*to reflux)*, leading to the formation of a white precipitate. The reaction mixture was stirred at room temperature for 3 h and then poured into water (100 mL). The layers were separated and the aqueous phase was further extracted with DCM (3 × 30 mL). The organic extracts were combined, dried over $MgSO_4$ and the solvent evaporated under reduced pressure. Residual methanesulfonylchloride was removed via co-evaporation with toluene under reduced pressure to obtain hexadecyl methanesulfonate as a waxy white solid in quantitative yield after drying under high vacuum. $^1H$ NMR (400 MHz, $CDCl_3$) δ (ppm) = 4.24 (t, *J* = 6.6 Hz, 2H), 3.02 (s, 3H), 1.81 – 1.72 (m, 2H), 1.46 – 1.22 (m, 26H), 0.90 (t, *J* = 6.8 Hz, 3H). It was used immediately without further purification.

*Reduction and alkylation*

Benzodithiophene-4-8-dione (500 mg, 2.27 mmol, 1.00 eq.), zinc powder (390 mg, 5.90 mmol, 2.60 eq.), sodium hydroxide pellets (1.36 g, 34.3 mmol, 15.1 eq.) and TBABr (380 mg, 0.70 mmol, 0.31 eq.) were combined in a round bottomed flask, which was subsequently evacuated and backfilled with argon 5 times. Degassed water (7 mL) was added to the mixture under argon, leading to a yellow suspension (*a slight exotherm can be observed as condensation on the flask walls)*. The temperature of the mixture was then raised to reflux resulting in a colour change, firstly to a deep red suspension and then to a biphasic mixture, with a dark brown upper layer and a clear faint orange bottom layer. The mixture was refluxed for 1.5 h. Freshly prepared hexadecyl methanesulfonate (2.91 g, 9.08 mmol. 4.00 eq.) was then quickly added to the refluxing solution through the condenser in one portion under a heavy argon flow, causing the reaction mixture to instantly turn black. It was then vigorously stirred at reflux overnight (*within ca. 15 min the black colour dissipated and the mixture was biphasic, with a dark brown upper layer and a milky white bottom layer*), cooled to room temperature, diluted with water (100 mL) and poured into DCM (100 mL). The layers were separated and the aqueous layer was further extracted with DCM (3 × 30 mL). The organic extracts were combined, dried over $MgSO_4$ and the solvent evaporated under reduced pressure. The residue was purified by column chromatography on silica gel (eluent: firstly *n*−hexane to elute an impurity and then 5:95 DCM/ *n*−hexane to elute the product) and then recrystallized from ethanol (50 mL, reflux to −20 °C) to obtain 4,8-di(hexadecyloxy)benzodithiophene (**S6a**) as colourless crystals (1.45 g, 2.16 mmol, 95%) that were sufficiently pure for the next step. $^1H$ NMR data were in accordance with those reported in the literature.[9] $^1H$ NMR (400 MHz, $CDCl_3$) δ (ppm) = 7.50 (d, *J* = 5.5 Hz, 2H), 7.39 (d, *J* = 5.5 Hz, 2H), 4.30 (t, *J* = 6.6 Hz, 4H), 1.94 – 1.85 (m, 4H), 1.64 – 1.54 (m, 4H), 1.46 – 1.22 (m, 48H), 0.90 (t, *J* = 6.8 Hz, 6H).

**4,8-Di(2-ethylhexyloxy)benzodithiophene** (**S6b**). Prepared analogously to (**S6a**) on the same scale. 2-Ethylhexyl methanesulfonate was isolated as a light yellow oil in quantitative yield. $^1H$ NMR (400 MHz, $CDCl_3$) δ (ppm) = 4.20 – 4.12 (m, 2H), 3.02 (s, 3H), 1.68 (dq, *J* = 12.2, 6.1 Hz, 1H), 1.48 – 1.26 (m, 8H), 0.98 – 0.89 (m, 6H). 4,8-Di(2-ethylhexyloxy)benzodithiophene (**S6b**) was purified by column chromatography on silica gel (eluent: firstly *n*−hexane to elute an impurity and then 5:95 DCM/ *n*−hexane to elute the product) and isolated as a clear oil (825 mg, 18.5 mmol, 81%). $^1H$ NMR data were in accordance with those reported in the literature.[9] $^1H$ NMR (400 MHz, $CDCl_3$) δ (ppm) = 7.50 (d, *J* = 5.5 Hz, 2H), 7.39 (d, *J* = 5.5 Hz, 2H), 4.24 – 4.16 (m, 4H), 1.88 – 1.35 (m, 18H), 1.04 (t, *J* = 7.4 Hz, 6H), 0.96 (t, *J* = 7.1 Hz, 6H).

**2,6-Bis(trimethylstannyl)-4,8-di(hexadecyloxy)benzodithiophene** (**S7a**). Prepared based on a literature procedure.[9] 4,8-Di(hexadecyloxy)benzodithiophene (**S6a**) (1.40 g, 2.09 mmol, 1.00 eq.) was dissolved in dry THF (75 mL) under argon and cooled to −78 °C in a dry ice bath, giving a white

suspension. *n*–BuLi (1.6 M in hexane, 3.14 mL, 5.02 mmol, 2.40 eq.) was added dropwise and the mixture was stirred at −78 °C for 1 h. The reaction was then removed from the dry ice bath and stirred at room temperature for 45 min. The reaction mixture was still a white suspension. It was then cooled back to −78 °C and trimethyltinchloride (1 M in THF, 5.64 mL, 5.64 mmol, 2.70 eq.) was added dropwise. The reaction was removed from the dry ice bath (*upon warming to room temperature it turned clear*). It was stirred at room temperature for 2 h and then poured into cool water (200 mL) and extracted with diethyl ether (3 × 100 mL). The organic extracts were combined, dried over $MgSO_4$ and the solvent removed under reduced pressure. The residue was recrystallized three times from ethanol/ *n*-hexane (60 mL ethanol, 10 mL *n*-hexane, reflux to −20 °C) to obtain 2,6-bis(trimethylstannyl)-4,8-di(hexadecyloxy)benzodithiophene (**S7a**) as fluffy colourless needles (1.17 g, 1.17 mmol, 56%). $^1$H NMR data were in accordance with those reported in the literature.[9] $^1$H NMR (400 MHz, $d_6$-benzene) δ (ppm) = 7.92 (s + (d, $^3J_{H–Sn}$ = 29.4 Hz), 2H), 4.42 (t, *J* = 6.5 Hz, 4H), 1.93 – 1.84 (m, 4H), 1.30 – 1.22 (m, 48H), 0.92 (t, *J* = 6.7 Hz, 6H), 0.32 (s, (d, $^2J_{H–Sn}$ = 56.3 Hz), 18H).

**2,6-Bis(trimethylstannyl)-4,8-di(2-ethylhexyloxy)benzodithiophene** (**S7b**). Prepared analogously to (**X**) starting from 4,8-di(2-ethylhexyloxy)benzodithiophene (**S7a**) (777 mg, 1.74 mmol). The crude product was recrystallized twice from ethanol (20 mL, reflux to −20 °C) to obtain 2,6-bis(trimethylstannyl)-4,8-di(2-ethylhexyloxy)benzodithiophene (**S7b**) as colourless crystals (750 mg, 0.97 mmol, 56%). $^1$H NMR data were in accordance with those reported in the literature.[9] $^1$H NMR (400 MHz, $d_6$-benzene) δ (ppm) = 7.92 (s + (d, $^3J_{H–Sn}$ = 29.2 Hz), 2H), 4.34 (dd, *J* = 5.2, 1.3 Hz, 4H), 1.84 – 1.33 (m, 18H), 1.02 (t, *J* = 7.3 Hz, 6H), 0.95 (t, *J* = 7.0 Hz, 6H), 0.32 (s, (d, $^2J_{H–Sn}$ = 56.3 Hz), 18H).

**General procedure for the synthesis of polymers.**[10] Polymerisations were carried out on a *ca.* 100 mg scale of the corresponding bis(stannane). The stannane monomer (1.00 eq.), palladium-5,15-di(4-bromophenyl)-$2^2$,$2^3$,$7^2$,$7^3$,$12^2$,$12^3$,$17^2$,$17^3$-octamethyltetrabenzoporphyrin (0.00 eq. for 0 mol%, 0.10 eq. for 5 mol%), tri(*ortho*-tolyl)phosphine (0.12 eq.) and bis(dibenzylideneacetone)palladium(0) (0.03 eq.) were combined in an oven and flame dried crimp cap 5 mL microwave vial under argon. Dry, degassed chlorobenzene (3 mL) was added immediately, followed by 2,5-dibromothiophene (**X**) (0.90 for 5 mol%, 1.00 eq. for 0 mol%), and the resulting solution (orange/ red for blanks, dark green for porphyrin-doped) was degassed for 5 min. The vial was then heated in a microwave reactor sequentially at 100 °C for 2 min, 125 °C for 2 min and finally at 150 °C for 1 h. The resulting dark red reaction mixture was added dropwise into vigorously stirring methanol (200 mL). The precipitate was filtered into a Soxhlet thimble and then washed in a Soxhlet extractor with acetone until the eluent ran clear (*note: while this may take ca. 2 h for the blank polymers, it was carried out overnight for porphyrin-doped polymers to ensure the removal of unreacted free porphyrin*). It was then washed with hexane for *ca.* 3–5 h until the eluent ran clear, and finally extracted with chloroform. The solvent volume was reduced to *ca.* 5 mL and the residue added dropwise into vigorously stirring methanol (200 mL) to precipitate the desired polymers, which were isolated via filtration and washed copiously with acetone.

**C16 0% Pd.** GPC (chlorobenzene) $M_n$ = 29,000, $M_w$ = 48,000, PDI = 1.7.

**C16 5% Pd.** GPC (chlorobenzene) $M_n$ = 14,000, $M_w$ = 31,000, PDI = 2.2.

**C2C4 0% Pd.** GPC (chlorobenzene) $M_n$ = 10,000, $M_w$ = 17,000 PDI = 1.7

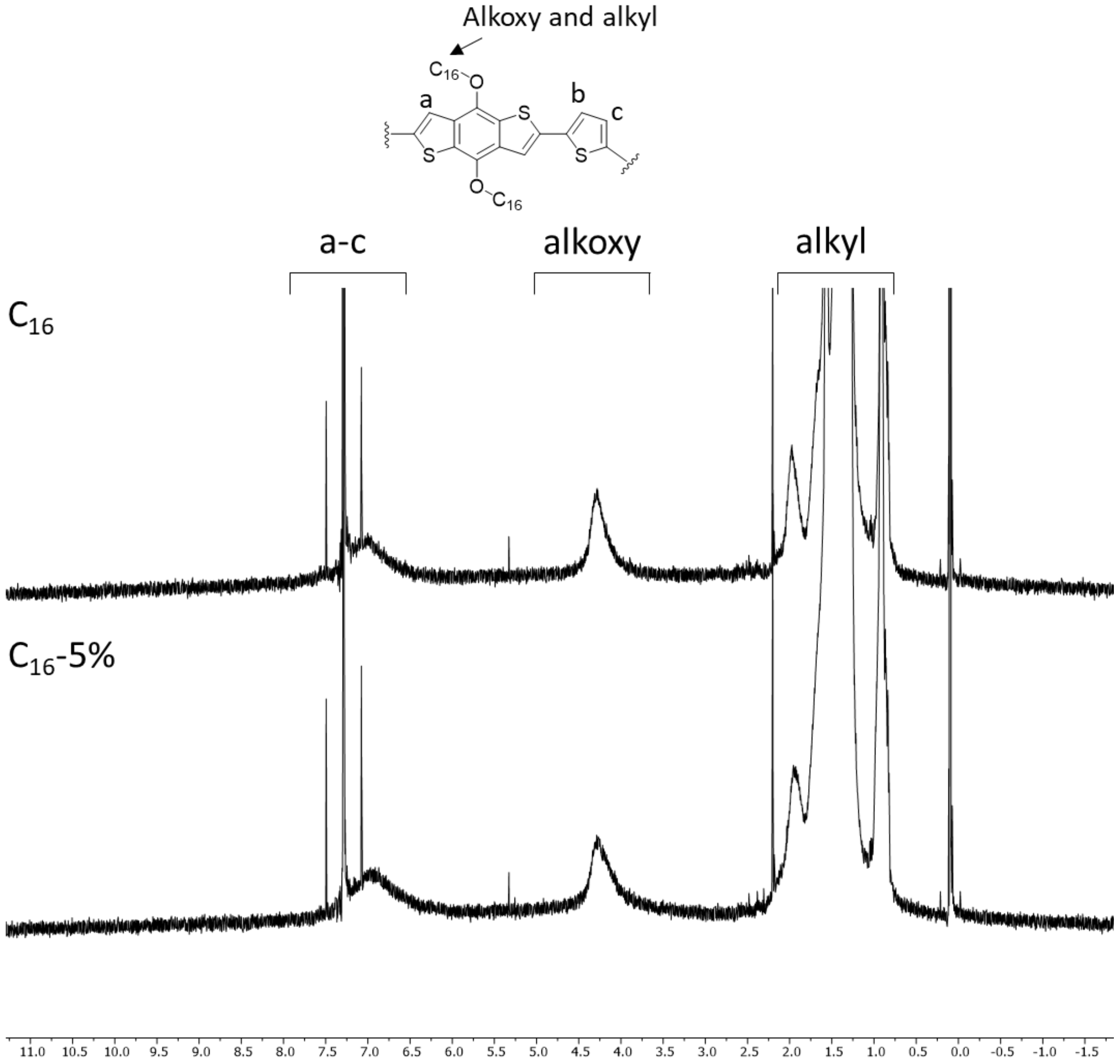


**NMR spectrum 5.** NMR spectra of $C_{16}$ and $C_{16}$-5% in $CDCl_3$.

**Spectroscopic analysis**

| Material | $\lambda_{abs}$ (nm) | Lifetime | Reference |
|---|---|---|---|
| Small Molecule | 250-365 | 0.1-2 s | 11 |
| Polymeric acrylamide derivatives | 280 | 0.5 s | 12 |
| Small Molecule solution | 350 | 30 ms | 13 |
| Polyacrylamide | 350 | 3 s | 14 |
| Small Molecule (with Br) | 350 | 0.6 ms | 15 |
| $PSSO_3H$ | 350 | 1 s | 16 |
| Small Molecule with Cl | 350 | 14 ms | 17 |
| Small Molecule | 380 | 36 ms | 18 |
| Carbon Dots- Mn | 420/520 | 10 ms | 19 |
| poly(phenyl-bithiophene)- Pt-porphyrin | 420 | < 2µs | 20 |
| Polymer, PSSO3 Na | 450 | 400 ms | 21 |
| Zn porphyrin in polymer matrix | 450 | 11 ms | 22 |
| Small Molecule Crystal | 450 | 300 ms | 23 |
| Rubrene | 530 | 100 µs | 24 |
| Small Molecule | 550 | ns range | 25 |
| Carbon Dots | 550 | 311 ms | 26 |
| Small molecule dispersed in Poly(methyl methacrylate) | 560 | 31 ms | 27 |
| Polymer, benzodithiophene + thiophene-1,1-dioxide | 500-600 | 1 µs | 28 |
| Bi-Bodipy | 629/530 | 4/60 µs | 29 |
| Polymer, Ra-P3HT | 420 | 5 µs | 30 |
| Polymer, F8BT | 460 | 180 µs | 31 |
| Co-polymer - F8BT–Zn porphyrin | 460 | 460 µs | 31 |
| Polymer, IF8TBTT | 540 | 1.3 µs | 30 |
| Polymer, APFO3 | 550 | 0.9 µs | 30 |
| Polymer, PCDTBT | 560 | 0.9 µs | 30 |
| Polymer, PBT7 | 690 | 0.8 µs | 30 |
| Polymer, IDT-BT | 660 | 0.1 µs | 30 |

**Table S1.** Example triplet lifetimes from the literature for small molecules and polymers with different bandgaps. The data reveals $C_2C_6$ and $C_{16}$-5% exhibit >2 orders of magnitude longer lifetimes compared to conjugated polymers and materials with similar bandgap.

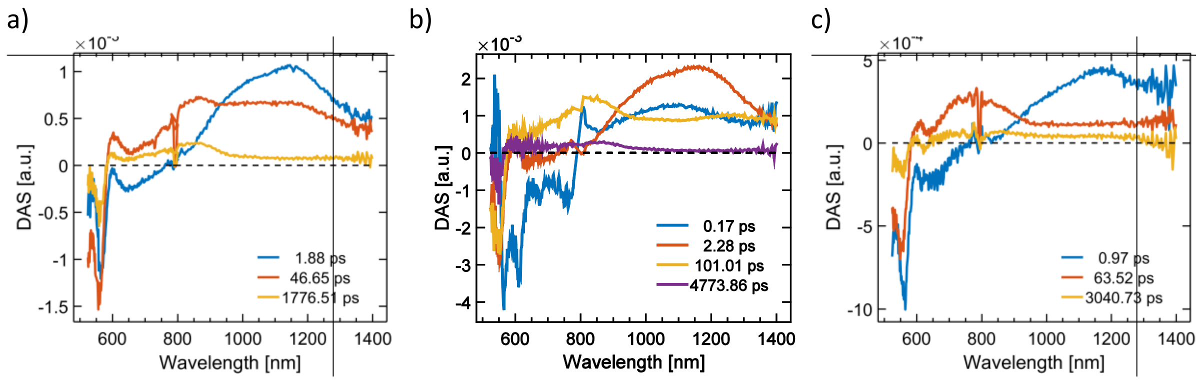


**Figure S1.** Decay associated spectra of **a)** $C_{16}$, **b)** $C_2C_5$ **c)** $C_{16}$-5% received from global fitting analysis performed with Optimus software of the ps-ns TA of the samples excited at 525 nm.

| | $C_{16}$ | $C_{16}$-5% |
|---|---|---|
| A1 (%) | 0.58(0.04) | 0.71(0.07) |
| τ1 (ps) | 1.6(0.1) | 0.6(0.2) |
| A2 (%) | 0.39(0.06) | 0.26(0.13) |
| τ2 (ps) | 50.4(0.2) | 25.9(0.3) |
| A3 (%) | 0.02(0.4) | 0.04(0.3) |
| τ3 (ps) | inf | inf |

**Table S2.** Time constants of the polymers singlet exciton ($S_1$) decay in $C_{16}$ and $C_{16}$-5% received from exponential fitting of the 1167 nm single wavelength kinetics extracted from the data in Figure 4a,b in the main text. Three exponential fit was used and the third component was set to infinity to account for the long-lived signal, shown to decay on the microsecond timescale. The weighted average of the first two time constants was used to estimate the quenching value of 65%. The values in the brackets correspond to the standard error estimated from the fits.

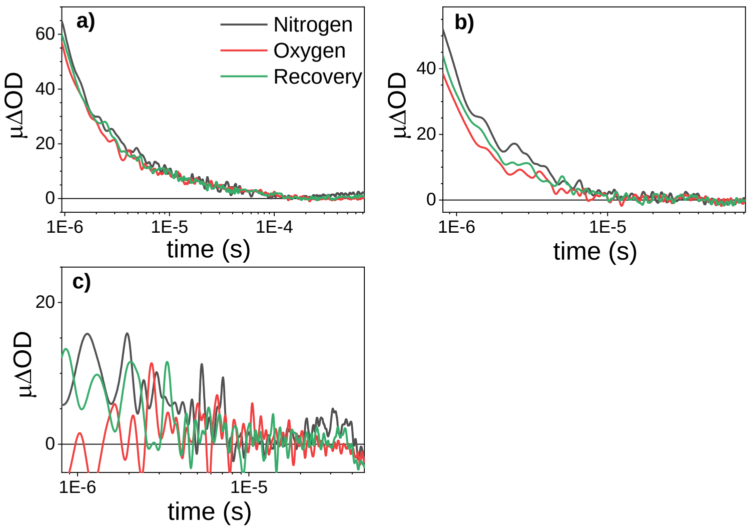


**Figure S2.** Transient absorption decay of $C_{16}$ polymer measured under nitrogen (black), oxygen (red) and back to nitrogen (green). The excitation was 520 nm and 30 μJ/cm$^2$ and the probe was **a)** 800, **b)** 950 and **c)** 1500 nm.

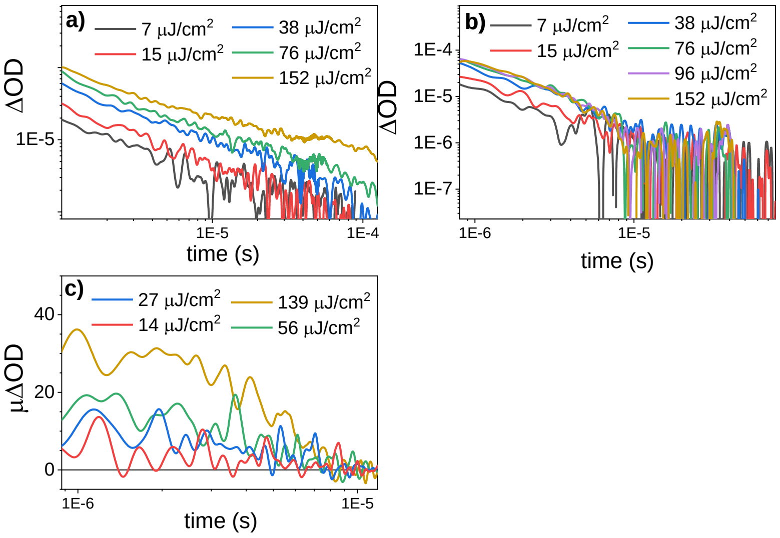


**Figure S3.** Transient absorption decay of $C_{16}$ probed at **a)** 800, **b)** 950 and **c)** 1500 nm. The decays were obtained exciting at 520 nm with an excitation density that goes from 7 to 150 μJ/cm$^2$.

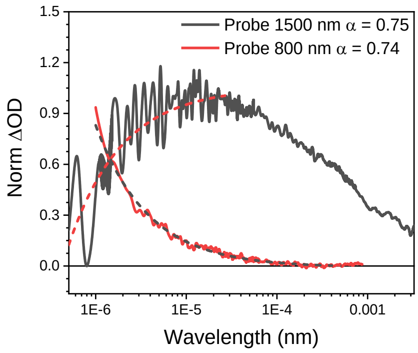


**Figure S4**. Normalized transient absorption decays of $C_2C_6$ polymer probed at 1500 (black) and 850 (red) nm. These decays were obtained exciting at 520 nm with an energy density of 28 μJ/cm$^2$. Dashed lines are fitting to power law.

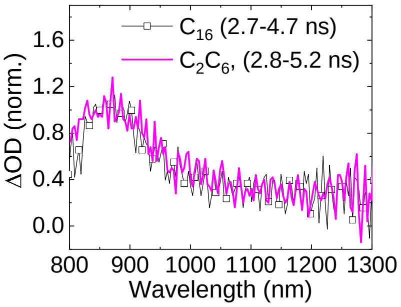


**Figure S5**. Normalized nanosecond transient absorption spectrum of $C_{16}$ (black with open squares) and $C_2C_6$ (purple line) extracted from ultrafast transient absorption spectra in Figure 2a and 2b, respectively.

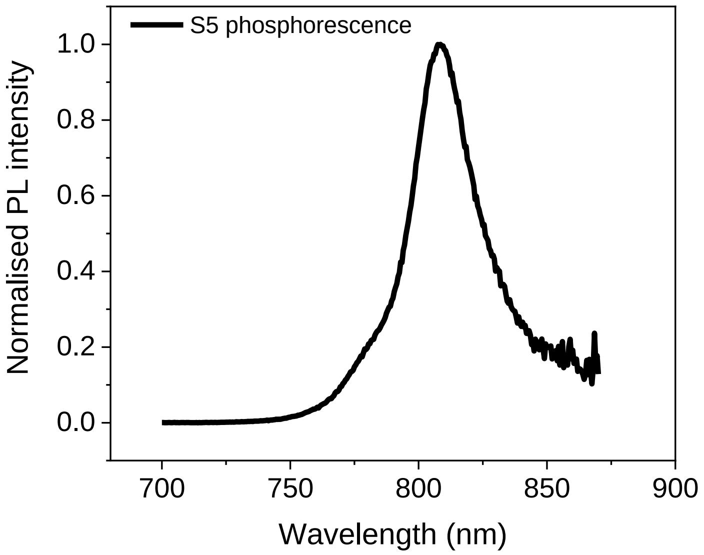


**Figure S6.** Phosphorescence spectrum of Pd-porphyrin solution in degassed dimethylformamide.

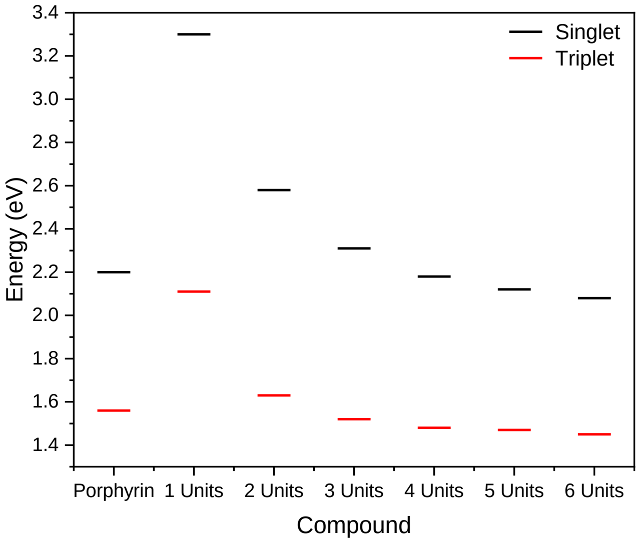


**Figure S7.** $S_1$ singlet and $T_1$ triplet excited states energy levels calculated for the Pd-porphyrin and BDT-Th oligomers up to the hexamer at TD-B3LYP/6-31G* (LANL2DZ pseudopotential for the Pd atom) used for designing $C_{16}$-5%.

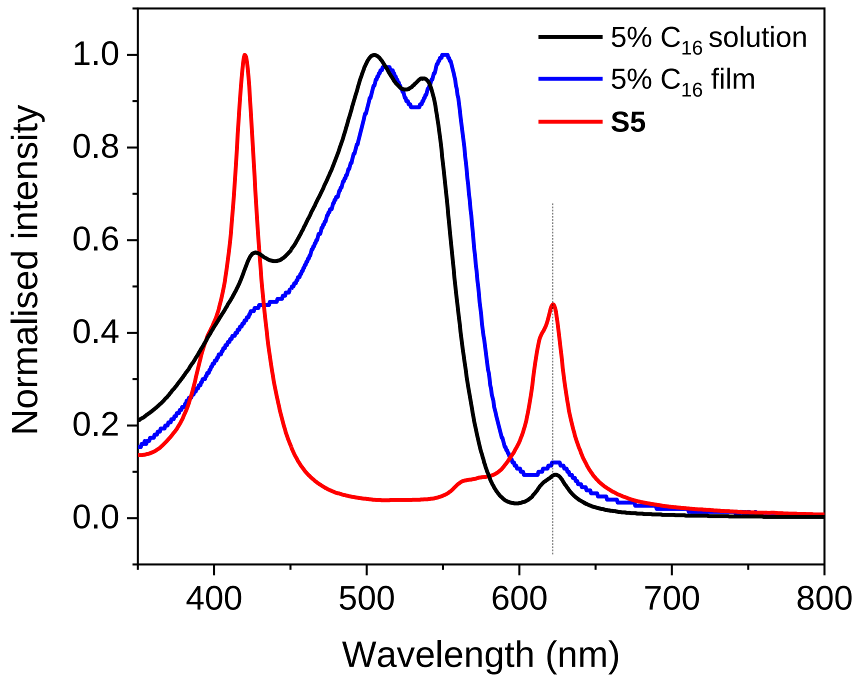


**Figure S8**. Absorption spectra for the 5% C16 polymer and the metalloporphyrin S5.

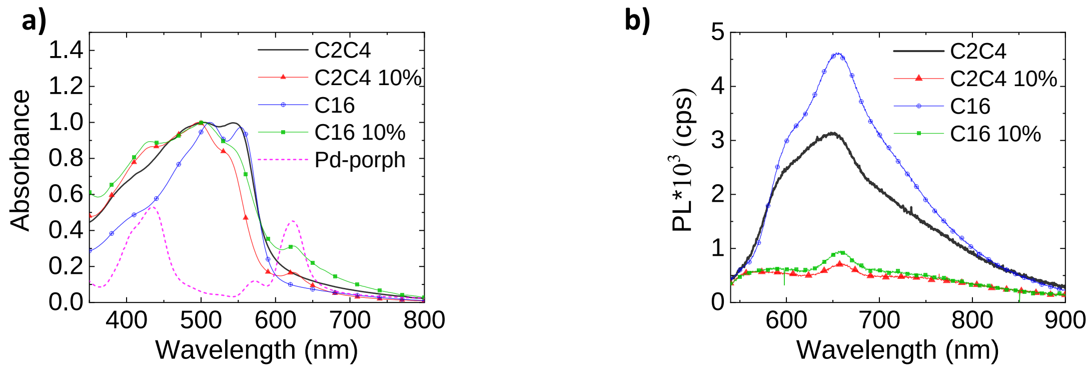


**Figure S9.** Steady-state **(a)** absorption and **(b)** fluorescence spectra of thin films. The films were spin-coated from chlorobenzene solutions.

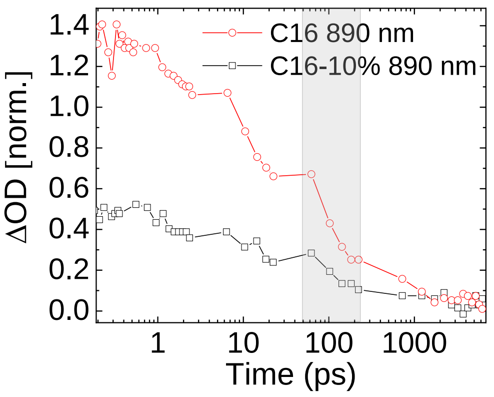


**Figure S10.** Transient absorption signal at 890 nm of $C_{16}$ and $C_{16}$-5% normalized for photons absorbed. The highlighted area was used to calculate the % drop in polaron signal in $C_{16}$-5% as shown in Table S2

| time (ps) | $C_{16}$ (OD) | $C_{16}$-5% (OD) | % drop |
|---|---|---|---|
| 63 | 2.84E-04 | 6.71E-04 | 58 |
| 103 | 1.94E-04 | 4.30E-04 | 55 |
| 143 | 1.34E-04 | 3.15E-04 | 57 |
| 183 | 1.34E-04 | 2.52E-04 | 47 |
| 223 | 1.05E-04 | 2.52E-04 | 58 |
| | | Average drop | 55 |
| | | Standard deviation of drop | 5 |

**Table S3.** Transient absorption signal amplitude at 890 nm in $C_{16}$ and $C_{16}$-5% after $S_1$ decay normalized for photons absorbed. The fourth column is the signal drop in $C_{16}$-5% as compared to $C_{16}$.

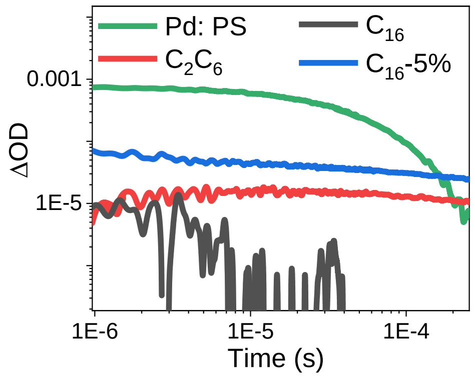


**Figure S11.** Transient absorption decays of triplet species of Pd-Porphyrin (green), $C_2C_6$ (red) $C_{16}$ (black) an $C_{16}$-5% (blue) probed. Pd-porphyrin:polystyrene film was excited and probed at 500 and 700 nm, respectively at 20 μJ/cm$^2$.

| **Compound** | **E ox onset(V)** | **E red onset(V)** | **HOMO (eV)** | **LUMO (eV)** | **S1 (eV)** | **P1 (eV)** |
|---|---|---|---|---|---|---|
| PdTPTBP | 0.70 | -1.2 | -5.42 | -3.52 | 1.91 | 1.56 |
| PBDT-T | 0.44 | -2.05 | -5.16 | -2.67 | 2.13 | |

**Table S4.** Electrochemistry data for PBDT-T and Pd-porphyrin from literature,[8,32] referenced to Ag/AgCl. $S_1$ of estimated from the crossing point of normalized absorbance and fluorescence spectrum.

## References


1. Waykole, L.; Paquette, L. A. Ethynyl P-Tolyl Sulfone. *Organic Syntheses* **1989**, *67*, 149. https://doi.org/10.15227/orgsyn.067.0149.
2. Otten, A.; Namyslo, J. C.; Stoermer, M.; Kaufmann, D. E. 2-(Het)Aryl-Substituted 7-Azabicyclo[2.2.1]Heptane Systems. *European J Org Chem* **1998**, *1998* (9), 1997–2001. https://doi.org/10.1002/(SICI)1099-0690(199809)1998:9<1997::AID-EJOC1997>3.0.CO;2-A.
3. Okujima, T.; Jin, G.; Hashimoto, Y.; Yamada, H.; Uno, H.; Ono, N. Synthesis of 4,7-Dihydro-2H-Isoindole Derivatives via Diels—Alder Reaction of Tosylacetylene. *ChemInform* **2007**, *38* (23). https://doi.org/10.1002/chin.200723101.
4. Filatov, M. A.; Cheprakov, A. v.; Beletskaya, I. P. A Facile and Reliable Method for the Synthesis of Tetrabenzoporphyrin from 4,7-Dihydroisoindole. *European J Org Chem* **2007**, *2007* (21), 3468–3475. https://doi.org/10.1002/ejoc.200700014.
5. Filatov, M. A.; Lebedev, A. Y.; Vinogradov, S. A.; Cheprakov, A. v. Synthesis of 5,15-Diaryltetrabenzoporphyrins. *J Org Chem* **2008**, *73* (11), 4175–4185. https://doi.org/10.1021/jo800509k.
6. Sommer, J. R.; Shelton, A. H.; Parthasarathy, A.; Ghiviriga, I.; Reynolds, J. R.; Schanze, K. S. Photophysical Properties of Near-Infrared Phosphorescent π-Extended Platinum Porphyrins. *Chemistry of Materials* **2011**, *23* (24), 5296–5304. https://doi.org/10.1021/cm202241e.
7. Andrianov, D. S.; Levitskiy, O. A.; Rybakov, V. B.; Magdesieva, T. v.; Cheprakov, A. v. Metal Complexes of Diaryltetrabenzodiazaporphyrins. *ChemistrySelect* **2016**, *1* (3), 360–374. https://doi.org/10.1002/slct.201600118.
8. Hou, J.; Park, M.-H.; Zhang, S.; Yao, Y.; Chen, L.-M.; Li, J.-H.; Yang, Y. Bandgap and Molecular Energy Level Control of Conjugated Polymer Photovoltaic Materials Based on Benzo[1,2- *b* :4,5- *b* ′]Dithiophene. *Macromolecules* **2008**, *41* (16), 6012–6018. https://doi.org/10.1021/ma800820r.
9. Labban, A.; Warnan, J.; Cabanetos, C.; Ratel, O.; Tassone, C.; Toney, M. F.; Beaujuge, P. M. Dependence of Crystallite Formation and Preferential Backbone Orientations on the Side Chain Pattern in PBDTTPD Polymers. *ACS Appl Mater Interfaces* **2014**, *6* (22), 19477–19481. https://doi.org/10.1021/am505280a.
10. Wolf, J.; Cruciani, F.; el Labban, A.; Beaujuge, P. M. Wide Band-Gap 3,4-Difluorothiophene-Based Polymer with 7% Solar Cell Efficiency: An Alternative to P3HT. *Chemistry of Materials* **2015**, *27* (12), 4184–4187. https://doi.org/10.1021/acs.chemmater.5b01520.
11. Guo, J.; Yang, C.; Zhao, Y. Long-Lived Organic Room-Temperature Phosphorescence from Amorphous Polymer Systems. *Acc Chem Res* **2022**, *55* (8), 1160–1170. https://doi.org/10.1021/acs.accounts.2c00038.
12. Ma, X.; Xu, C.; Wang, J.; Tian, H. Amorphous Pure Organic Polymers for Heavy-Atom-Free Efficient Room-Temperature Phosphorescence Emission. *Angewandte Chemie International Edition* **2018**, *57* (34), 10854–10858. https://doi.org/10.1002/anie.201803947.
13. Bhatia, H.; Ray, D. Use of Dimeric Excited States of the Donors in $D_4$-A Systems for Accessing White Light Emission, Afterglow, and Invisible Security Ink. *The Journal of Physical Chemistry C* **2019**, *123* (36), 22104–22113. https://doi.org/10.1021/acs.jpcc.9b07762.

14. Zhang, Y.; Liu, C.; Zhen, H.; Lin, M. Microwave-Assisted Establishment of Efficient Amorphous Polymeric Phosphorescent Materials with Ultralong Blue Afterglow. *J Mater Chem C Mater* **2021**, *9* (15), 5277–5288. https://doi.org/10.1039/D0TC05171C.
15. Fateminia, S. M. A.; Mao, Z.; Xu, S.; Yang, Z.; Chi, Z.; Liu, B. Organic Nanocrystals with Bright Red Persistent Room-Temperature Phosphorescence for Biological Applications. *Angewandte Chemie International Edition* **2017**, *56* (40), 12160–12164. https://doi.org/10.1002/anie.201705945.
16. Ogoshi, T.; Tsuchida, H.; Kakuta, T.; Yamagishi, T.; Taema, A.; Ono, T.; Sugimoto, M.; Mizuno, M. Ultralong Room-Temperature Phosphorescence from Amorphous Polymer Poly(Styrene Sulfonic Acid) in Air in the Dry Solid State. *Adv Funct Mater* **2018**, *28* (16), 1707369. https://doi.org/10.1002/adfm.201707369.
17. Ma, L.; Sun, S.; Ding, B.; Ma, X.; Tian, H. Highly Efficient Room-Temperature Phosphorescence Based on Single-Benzene Structure Molecules and Photoactivated Luminescence with Afterglow. *Adv Funct Mater* **2021**, *31* (17), 2010659. https://doi.org/10.1002/adfm.202010659.
18. Liao, F.; Du, J.; Nie, X.; Wu, Z.; Su, H.; Huang, W.; Wang, T.; Chen, B.; Jiang, J.; Zhang, X.; Zhang, G. Modulation of Red Organic Room-Temperature Phosphorescence in Heavy Atom-Free Phosphors. *Dyes and Pigments* **2021**, *193*, 109505. https://doi.org/10.1016/j.dyepig.2021.109505.
19. Wang, B.; Yu, Y.; Zhang, H.; Xuan, Y.; Chen, G.; Ma, W.; Li, J.; Yu, J. Carbon Dots in a Matrix: Energy-Transfer-Enhanced Room-Temperature Red Phosphorescence. *Angewandte Chemie International Edition* **2019**, *58* (51), 18443–18448. https://doi.org/10.1002/anie.201911035.
20. Andernach, R.; Utzat, H.; Dimitrov, S. D.; McCulloch, I.; Heeney, M.; Durrant, J. R.; Bronstein, H. Synthesis and Exciton Dynamics of Triplet Sensitized Conjugated Polymers. *J Am Chem Soc* **2015**, *137* (32), 10383–10390. https://doi.org/10.1021/jacs.5b06223.
21. Cai, S.; Ma, H.; Shi, H.; Wang, H.; Wang, X.; Xiao, L.; Ye, W.; Huang, K.; Cao, X.; Gan, N.; Ma, C.; Gu, M.; Song, L.; Xu, H.; Tao, Y.; Zhang, C.; Yao, W.; An, Z.; Huang, W. Enabling Long-Lived Organic Room Temperature Phosphorescence in Polymers by Subunit Interlocking. *Nat Commun* **2019**, *10* (1), 4247. https://doi.org/10.1038/s41467-019-11749-x.
22. Durandin, N. A.; Isokuortti, J.; Efimov, A.; Vuorimaa-Laukkanen, E.; Tkachenko, N. v.; Laaksonen, T. Efficient Photon Upconversion at Remarkably Low Annihilator Concentrations in a Liquid Polymer Matrix: When Less Is More. *Chemical Communications* **2018**, *54* (99), 14029–14032. https://doi.org/10.1039/C8CC07592A.
23. Yang, G.; Lv, A.; Xu, Z.; Song, Z.; Shen, K.; Lin, C.; Niu, G.; Ma, H.; Shi, H.; An, Z. Modulating the Triplet Chromophore Environment to Prolong the Emission Lifetime of Ultralong Organic Phosphorescence. *J Mater Chem C Mater* **2022**, *10* (37), 13747–13752. https://doi.org/10.1039/D2TC00836J.
24. Ryasnyanskiy, A.; Biaggio, I. Triplet Exciton Dynamics in Rubrene Single Crystals. *Phys Rev B* **2011**, *84* (19), 193203. https://doi.org/10.1103/PhysRevB.84.193203.
25. Shaikh, J.; Congrave, D. G.; Forster, A.; Minotto, A.; Cacialli, F.; Hele, T. J. H.; Penfold, T. J.; Bronstein, H.; Clarke, T. M. Intrinsic Photogeneration of Long-Lived Charges in a Donor-Orthogonal Acceptor Conjugated Polymer. *Chem Sci* **2021**, *12* (23), 8165–8177. https://doi.org/10.1039/D1SC00919B.
26. Cao, Q.; Liu, K.-K.; Liang, Y.-C.; Song, S.-Y.; Deng, Y.; Mao, X.; Wang, Y.; Zhao, W.-B.; Lou, Q.; Shan, C.-X. Brighten Triplet Excitons of Carbon Nanodots for Multicolor

Phosphorescence Films. *Nano Lett* **2022**, *22* (10), 4097–4105. https://doi.org/10.1021/acs.nanolett.2c00788.

27. Kuila, S.; Ghorai, A.; Samanta, P. K.; Siram, R. B. K.; Pati, S. K.; Narayan, K. S.; George, S. J. Red-Emitting Delayed Fluorescence and Room Temperature Phosphorescence from Core-Substituted Naphthalene Diimides. *Chemistry – A European Journal* **2019**, *25* (70), 16007–16011. https://doi.org/10.1002/chem.201904651.
28. Busby, E.; Xia, J.; Wu, Q.; Low, J. Z.; Song, R.; Miller, J. R.; Zhu, X.-Y.; Campos, L. M.; Sfeir, M. Y. A Design Strategy for Intramolecular Singlet Fission Mediated by Charge-Transfer States in Donor–Acceptor Organic Materials. *Nat Mater* **2015**, *14* (4), 426–433. https://doi.org/10.1038/nmat4175.
29. Wu, W.; Guo, H.; Wu, W.; Ji, S.; Zhao, J. Organic Triplet Sensitizer Library Derived from a Single Chromophore (BODIPY) with Long-Lived Triplet Excited State for Triplet–Triplet Annihilation Based Upconversion. *J Org Chem* **2011**, *76* (17), 7056–7064. https://doi.org/10.1021/jo200990y.
30. Soon, Y. W.; Shoaee, S.; Ashraf, R. S.; Bronstein, H.; Schroeder, B. C.; Zhang, W.; Fei, Z.; Heeney, M.; McCulloch, I.; Durrant, J. R. Material Crystallinity as a Determinant of Triplet Dynamics and Oxygen Quenching in Donor Polymers for Organic Photovoltaic Devices. *Adv Funct Mater* **2014**, *24* (10), 1474–1482. https://doi.org/10.1002/adfm.201302612.
31. Shaikh, J.; Freeman, D. M. E.; Bronstein, H.; Clarke, T. M. Energy-Transfer Pathways and Triplet Lifetime Manipulation in a Zinc Porphyrin/F8BT Hybrid Polymer. *The Journal of Physical Chemistry C* **2018**, *122* (42), 23950–23958. https://doi.org/10.1021/acs.jpcc.8b07880.
32. Shen, Z.; Zheng, S.; Xiao, S.; Shen, R.; Liu, S.; Hu, J. Red-Light-Mediated Photoredox Catalysis Enables Self-Reporting Nitric Oxide Release for Efficient Antibacterial Treatment. *Angewandte Chemie International Edition* **2021**, *60* (37), 20452–20460. https://doi.org/10.1002/anie.202107155.
